# Air-stable sodium dimethylglyoxime as a cathode presodiation additive for high-energy-density sodium-ion batteries

**DOI:** 10.1039/d6sc04167a

**Published:** 2026-06-30

**Authors:** Zhibin Zhao, Linxin Lv, Xinlei Xu, Jincheng Sun, Yifan Tong, Kai Zhang, Weiwei Huang

**Affiliations:** a Hebei Key Laboratory of Applied Chemistry, Yanshan University Qinhuangdao 066000 P. R. China huangweiwei@ysu.edu.cn; b Frontiers Science Center for New Organic Matter, Key Laboratory of Advanced Energy Materials Chemistry (Ministry of Education), State Key Laboratory of Advanced Chemical Power Sources, College of Chemistry, Nankai University Tianjin 300071 P. R. China; c College of Chemistry, Tianjin Normal University Tianjin 300071 P. R. China

## Abstract

Cathode presodiation has emerged as a crucial strategy to address the initial sodium loss that severely limits the initial coulombic efficiency and energy density of sodium-ion batteries. However, existing presodiation additives suffer from intrinsic drawbacks including poor air stability, mismatched decomposition potentials, and undesirable gas evolution which critically hinder their practical application. Herein, we present sodium dimethylglyoxime (Na_2_DMGO). By structurally translating oxime–lithium into a sodium-based salt, we cleverly leverage its inherent solubility as a unique advantage, delivering a robust and gas-free presodiation additive. Na_2_DMGO delivers a sodium release capacity of 330 mAh g^−1^ and decomposes at 3.4 V. *In situ* Fourier-transform infrared spectroscopy and differential electrochemical mass spectrometry analyses confirm its complete, gas-free decomposition into non-toxic DMODO *via* a C=N bond cleavage pathway. When 5 wt% Na_2_DMGO is incorporated into a Na_3_V_2_(PO_4_)_3_ cathode and paired with a hard carbon anode, the capacity retention improves from 62% to 85% and this improvement is accompanied by an increase in energy density from 117 to 131.5 Wh kg^−1^ (based on the mass of the active material only). These results highlight Na_2_DMGO as a practical and scalable sodium-supplementing additive that effectively mitigates irreversible Na^+^ loss.

## Abstract 2

Sacrificial sodium dimethylglyoxime (Na_2_DMGO) delivers 330 mAh g^−1^*via* gas-free decomposition, boosting full-cell capacity retention from 62% to 85% and enhancing energy density by 12.4% (from 117 to 131.5 Wh kg^−1^).

## Graphical abstract

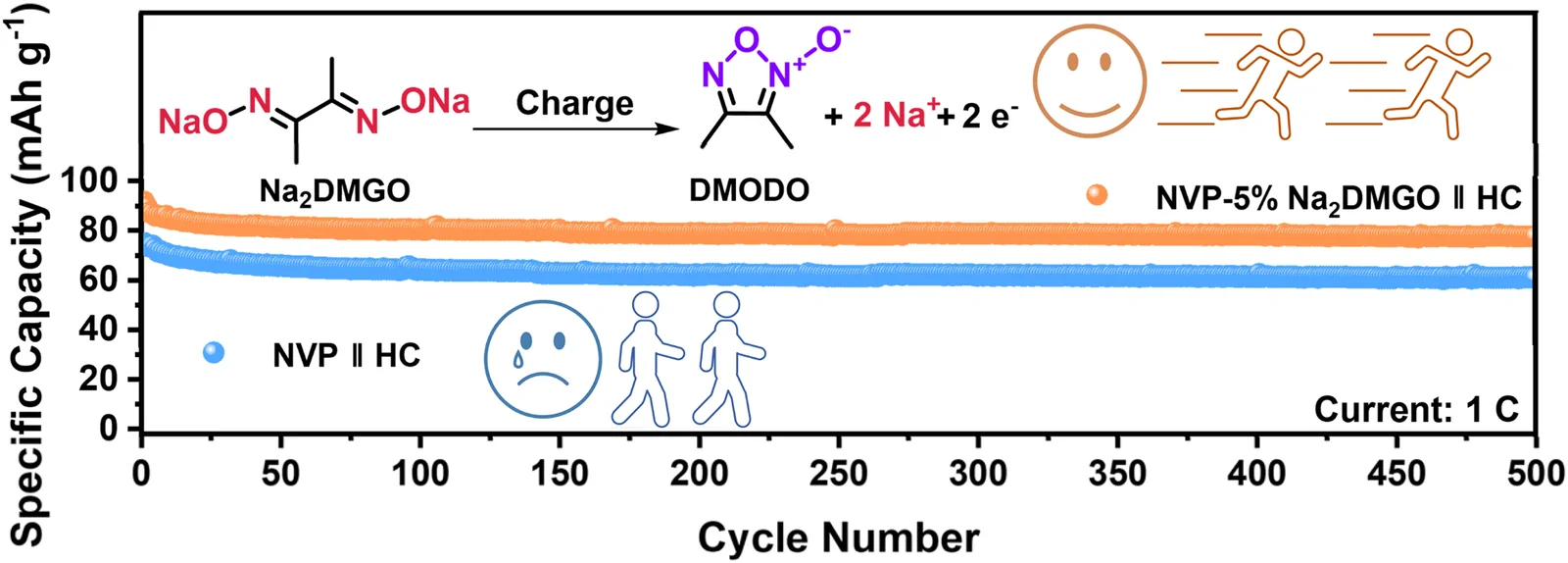

## Introduction

In practical sodium-ion full cells, all active Na^+^ needs to be provided by the cathode. Yet, substantial active Na^+^ loss occurs during the first cycle due to interfacial side reactions especially in solid electrolyte interface (SEI) formation at the anode, leading to low initial coulombic efficiency (ICE) and energy loss.^1–3^ Therefore, developing practical strategies to compensate for Na^+^ loss is crucial for improving energy density and cycle life in sodium ion batteries (SIBs).^4–6^ In recent years, the addition of a presodiation additive has gained attention as an effective approach to mitigate initial sodium loss.^7–9^ An ideal presodiation additive requires not only high sodium content but also a suitable redox potential and sufficient stability; however, simultaneously achieving electrochemical potential compatibility with cathode materials while maintaining ambient air stability remains a formidable challenge, highlighting the urgent need for robust cathode-based sodium compensation strategies.^10^

Cathode presodiation additives for SIBs can generally be divided into inorganic and organic compounds. Although various inorganic additives have been extensively investigated (*e.g.*, Na_3_P,^11^ NaN_3_,^12^ NaNO_3_,^13^ NaCrO_2_,^14^ and Na_2_S,^15^), their practical application is severely hindered by inherent flammability, toxicity, and detrimental side reactions. In contrast, organic salts particularly sodium alkoxides and sodium carboxylates have garnered considerable attention owing to their structural simplicity, facile synthesis, and environmental benignity.^16–18,33^ They are often affected by undesirable gas evolution in decomposition, poor air/moisture stability, and incompatibility in decomposition potential-either too low (sodium alkoxides <3.0 V) or too high (sodium carboxylates >4.0 V). Consequently, the development of practical organic presodiation additives still faces a fundamental challenge; it remains difficult to simultaneously achieve an appropriate decomposition potential and sufficient air/moisture stability. This trade-off between potential compatibility and stability represents the key bottleneck preventing their wider application.

The immense structural tunability of organic compounds offers a viable solution to this bottleneck. By employing rational molecular engineering strategies, the redox potentials and decomposition kinetics of these additives can be systematically optimized. Previous studies on lithium-ion batteries (LIBs) have demonstrated that introducing oxime groups can significantly elevate the redox potential (∼3.0 V), surpassing that of alkoxides (<3.0 V), C=O (∼2.3 V), and C=N (∼2.2 V).^19^ Nevertheless, oxime lithium compounds suffer from excessive solubility in organic electrolytes, which leads to their rapid dissolution and undermines their direct use as electrode materials. Transforming oxime–lithium into sodium-based salts represents a promising strategy. The inherently high solubility of oxime–sodium salts can be strategically harnessed as a unique advantage for controlled sodium release, which is further augmented by their chemical robustness and facile synthesis.^20,21^ Such oxime–sodium compounds therefore hold strong potential as practical presodiation additives. To the best of our knowledge, this concept remains unexplored, warranting further investigation.

In this work, we report for the first time the use of sodium dimethylglyoxime (Na_2_DMGO) as a high-capacity, air-stable, and gas-free organic presodiation additive. Na_2_DMGO exhibits a remarkable sodium-release capacity of 330 mAh g^−1^ approaching its theoretical capacity of 335 mAh g^−1^. Furthermore, it efficiently releases Na^+^ through electrochemical decomposition at 3.4 V, falling safely within the operating potential window of SIBs. Full cells assembled with Na_3_V_2_(PO_4_)_3_ (NVP) cathodes and hard carbon (HC) anodes were evaluated with the addition of 3–10 wt% Na_2_DMGO, effectively compensated for the Na^+^ loss at the anode, thereby significantly enhancing the CE and cycling stability. In addition, we employed *in situ* Fourier-transform infrared spectroscopy (FT-IR) and mass spectrometry (MS) to systematically investigate the decomposition process of Na_2_DMGO. For the first time, we directly captured its structural evolution and identified the corresponding decomposition products, thereby elucidating the Na^+^ release mechanism of Na_2_DMGO. Compared with previously reported organic additives, Na_2_DMGO simultaneously achieves a favorable redox potential, intrinsic air stability, and clean decomposition behavior. This design concept cleverly repurposes the high solubility of oxime compounds into a key functional advantage that enables efficient and uniform sodium release throughout the electrode.

## Results and discussion

**Fig. 1:**
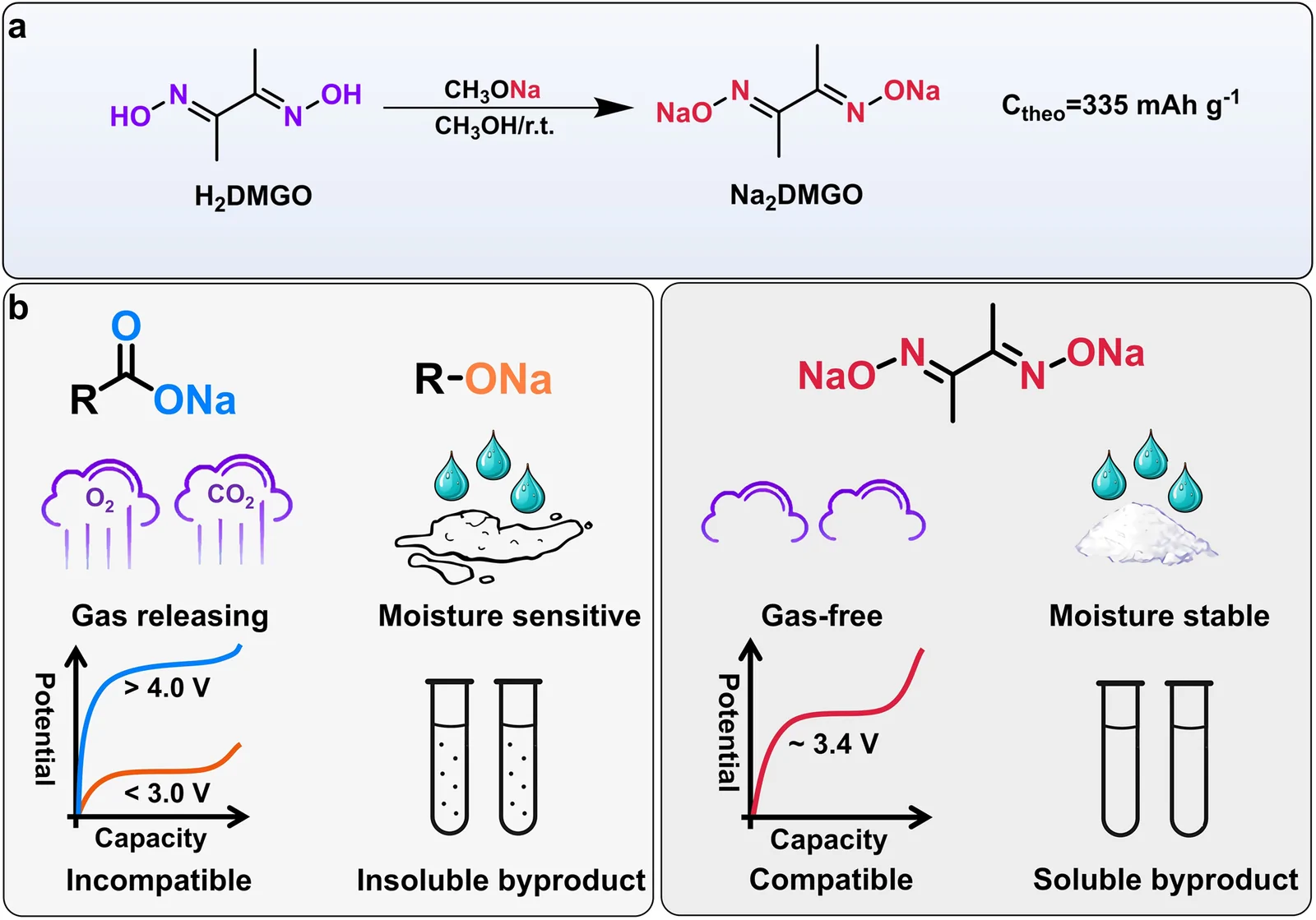
(a) Synthesis diagram of Na_2_DMGO. (b) Evolution of the organic presodiation additive toward safe, stable, and electrode-compatible presodiation systems.

A new organic presodiation additive, sodium dimethylglyoxime (Na_2_DMGO), was successfully synthesized *via* a one-step reaction between dimethylglyoxime (hereafter H_2_DMGO, to distinguish it from Na_2_DMGO) and CH_3_ONa at room temperature in methanol (Fig. 1a). Compared to conventional sodium alkoxide and carboxylate additives, Na_2_DMGO features a unique oxime-based structure, a decomposition potential well matched with that of typical cathodes, and high air and thermal stability. Upon decomposition, it generates soluble byproducts without gas evolution (Fig. 1b). The proposed mechanism reveals the sacrificial role of the additive in compensating for the initial sodium loss (Fig. S1). The structural and chemical characterization of Na_2_DMGO was systematically conducted using ^1^H nuclear magnetic resonance (NMR) spectroscopy, FT-IR spectroscopy, Raman spectroscopy, powder X-ray diffraction (PXRD), and X-ray photoelectron spectroscopy (XPS). The ^1^H NMR analysis (Fig. S2 and S3) highlights significant structural changes upon the conversion of H_2_DMGO to Na_2_DMGO. The appearance of the characteristic methyl resonance (–CH_3_) unambiguously confirms the successful preparation of Na_2_DMGO. The FT-IR spectra of H_2_DMGO and Na_2_DMGO (Fig. S4) are characterized by distinct peaks at 1589 cm^−1^, originating from the C=N bonds.^22^ Notably, a characteristic absorption peak observed at 3240 cm^−1^ in H_2_DMGO, attributable to O–H bonds, is absent in the FT-IR spectrum of Na_2_DMGO.^23^ Raman spectroscopic analysis of H_2_DMGO and Na_2_DMGO (Fig. S5) displays prominent peaks at approximately 1000 and 1300 cm^−1^, corresponding to C–C and C=N bonds. The characteristic absorption peak at 3140 cm^−1^, assigned to the O–H stretching vibration, completely disappears upon the sodiation reaction. PXRD analysis of H_2_DMGO and Na_2_DMGO uncovered distinct characteristic diffraction peaks, uncovering that the synthesized Na_2_DMGO possesses high purity and crystallinity (Fig. S6). The exceptional thermal stability of Na_2_DMGO is corroborated by thermogravimetric analysis (TGA), verifying a marginal mass loss of only 0.6% at 100 °C and a further negligible decline of 0.2% at 200 °C (Fig. S7). Density of states (DOS) calculations demonstrate that Na_2_DMGO exhibits a bandgap of less than 1.0 eV (Fig. S8). The calculated narrow band gap reflects the ionic nature of Na_2_DMGO rather than high electronic conductivity. Like most organic sacrificial additives, Na_2_DMGO relies on the conductive carbon matrix in the electrode for efficient electron transport. XPS was used to characterize the elemental composition and chemical states of Na_2_DMGO (Fig. S9). The O 1s spectrum of Na_2_DMGO has characteristic peaks at 532.2 eV (O–N) and 537.3 eV (Na–C–O), while the Na 1s peak at 1072.2 eV corresponds to Na–O, proving the successful synthesis of Na_2_DMGO.^24–26^ The scanning electron microscopy (SEM) images of Na_2_DMGO depict a sheet-like morphology (Fig. S10). Moreover, energy-dispersive X-ray spectroscopy (EDS) elemental mapping proves a homogeneous distribution of C, N, O, and Na throughout Na_2_DMGO.

Building on the above results, the air stability of Na_2_DMGO was systematically investigated under rigorously controlled conditions (25 °C and 40 °C, 25% relative humidity) for various durations. NMR spectroscopy (Fig. S11) illustrates the ^1^H NMR spectrum of Na_2_DMGO, with characteristic signals at *δ* = 2.3 ppm (–CH_3_), *δ* = 3.3 ppm (MeOH), and *δ* = 4.8 ppm (H_2_O). These peaks remain unchanged after air exposure, highlighting good structural stability. The exceptional air stability of Na_2_DMGO is further corroborated by MS (Fig. S12), where the molecular ion peak (*m*/*z* = 161[M + H]^+^) displays no signs of degradation after exposure to air for 1 h at 25 °C, or even after extended exposure for 1 day at 40 °C. The C=N (1589 cm^−1^) absorption peaks of Na_2_DMGO remain distinct and unaltered, even after prolonged exposure at 25 °C as shown in the FT-IR spectra (Fig. S13). Raman spectroscopy was used to gain deeper insights into the structural stability of Na_2_DMGO (Fig. S14). Na_2_DMGO retains structural characteristics closely resembling those of the pristine material, even with prolonged air exposure. Regarding the variation in the intensity of the Raman band at ∼3000 cm^−1^, this band corresponds to the standard C–H stretching vibrations of the methyl (–CH_3_) groups inherent to the pristine Na_2_DMGO structure. Crucially, the positions and sharpness of the characteristic skeletal vibration peaks (such as the C=N bond at ∼1589 cm^−1^) remain entirely consistent and unchanged across all conditions. Remarkably, this stability was uniformly observed under both temperature conditions (25 °C and 40 °C). XRD patterns (Fig. S15) further substantiate that the core structure and crystallinity of Na_2_DMGO remain exceptionally well-preserved under these conditions. Notably, a day of exposure at 40 °C enhanced the crystallinity of Na_2_DMGO and sequential photographs of the powder (Fig. S16) display no discernible changes in appearance, and the initial mass remained stable even after extended air exposure. These results demonstrate that Na_2_DMGO maintains its structural integrity upon air exposure, validating its remarkable ambient stability and practical viability as a presodiation additive.

**Fig. 2:**
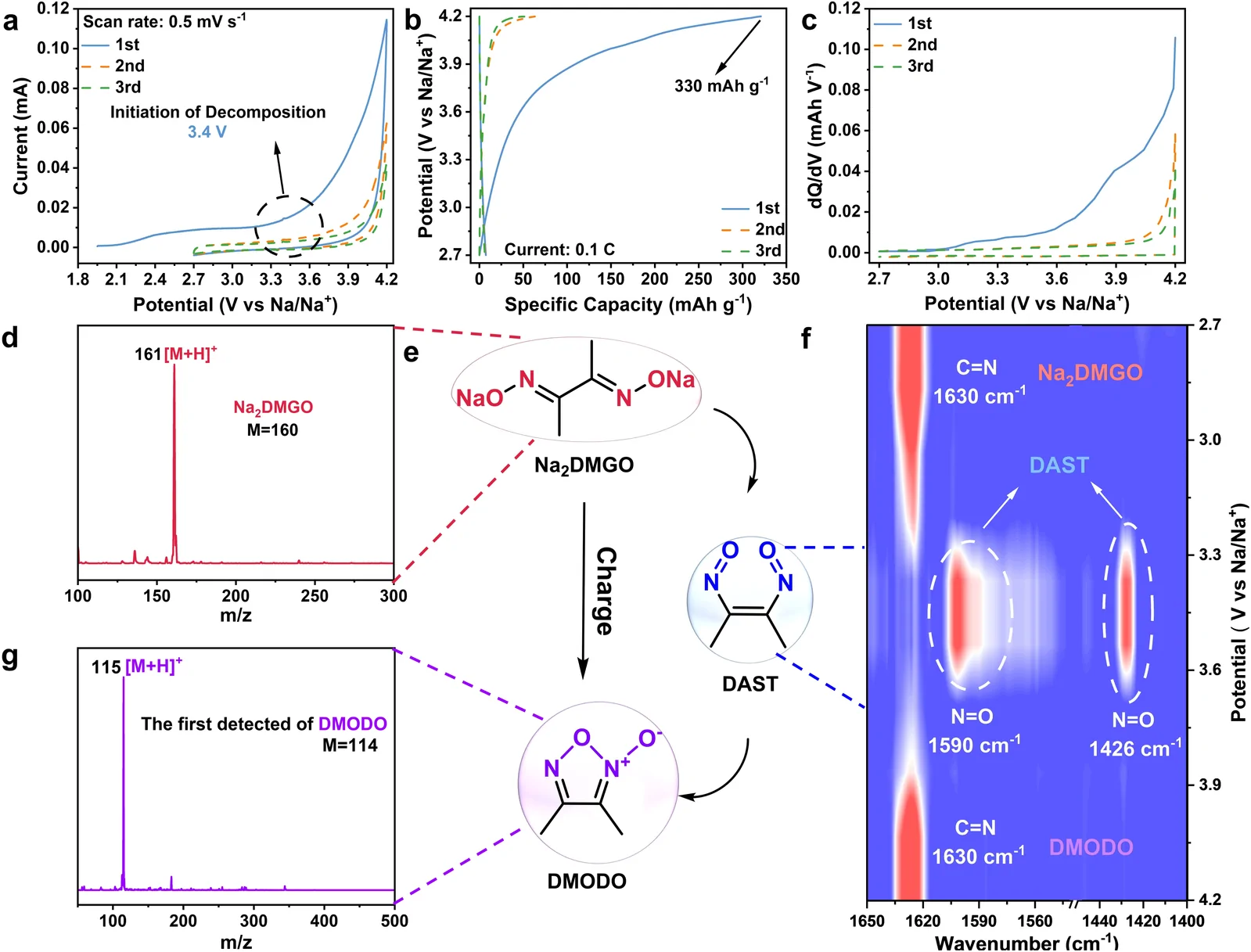
(a) CV curves for the Na_2_DMGO electrode. (b) Charge/discharge curves for the Na_2_DMGO electrode. (c) Differential capacity curve of the Na_2_DMGO electrode. (d) MS of Na_2_DMGO. (e) Schematic depiction of the Na_2_DMGO decomposition mechanism. (f) *In situ* FT-IR spectra of Na_2_DMGO during charge. (g) MS of DMODO.

The synthesized Na_2_DMGO was incorporated into a half-cell to evaluate its efficiency as a presodiation additive. Cyclic voltammetry (CV) was conducted to examine the electrochemical behavior of Na_2_DMGO. In the initial anodic scan, Na_2_DMGO decomposition begins at 3.4 V, peaks at 4.1 V, and completes Na^+^ release at approximately 4.2 V (Fig. 2a). Na_2_DMGO indicates a desodiation potential range that is well-suited for compatibility with the standard Na_3_V_2_(PO_4_)_3_ (NVP) electrode, thereby facilitating the complete release of active Na^+^ within the operational potential window of NVP. The absence of redox peaks in subsequent CV cycles provides further evidence of the irreversible nature of Na_2_DMGO decomposition. At a current rate of 0.1C (1C = 335 mAh g^−1^), the Na_2_DMGO half-cell delivered a substantial initial charge capacity of 330 mAh g^−1^ (Fig. 2b). This represents a decomposition rate of over 80%, while the subsequent discharge response remained trivial, highlighting the irreversible character of the additive. These results confirm that Na_2_DMGO releases considerable Na^+^ during the initial charging cycle, while consuming negligible sodium during the subsequent discharge. The differential capacity curve (Fig. 2c) was analyzed to further validate the sodium release process. For comparison, we tested the precursor dimethylglyoxime (H_2_DMGO) as an electrode material. H_2_DMGO displays low conductivity and delivers negligible capacity in battery tests, with CV curves exhibiting minimal current response and no distinct redox peaks, GCD curves indicating near-zero specific capacity, and electrochemical impedance spectroscopy (EIS) curves revealing high impedance (Fig. S17). A distinct desodiation peak appears exclusively in the first cycle and vanishes in subsequent cycles. Moreover, Na_2_DMGO retains its characteristic decomposition potential (∼3.4 V) and delivers a charge capacity of 327 mAh g^−1^ even after exposure to air for 1 day at 40 °C, manifesting its high stability exposed to air (Fig. S18). Collectively, these electrochemical results confirm that Na_2_DMGO functions as a high-efficiency and irreversible presodiation additive, well-suited for mitigating sodium loss in SIBs.

The elucidated decomposition mechanism demonstrates the structural transformation of Na_2_DMGO into DMODO upon sodium extraction (Fig. 2e). The structural and chemical characterization of the Na_2_DMGO decomposition mechanism was further investigated in-depth using *in situ* FT-IR and MS; the peak of Na_2_DMGO at 161 ([M + H]^+^) was observed, manifesting successful molecular ionization (Fig. 2d). Notably, the N=O double bond was characterized *via in situ* FT-IR, thereby underscoring the presence of the DAST intermediate. Specifically, the broad absorption peak at 1630 cm^−1^ in the spectrum gradually diminishes during the desodiation process, signifying the conversion of C=N bonds to C–N bonds; the re-emergence of signals in the C=N region is attributed to the stable DMODO (Fig. 2f). This bond transformation indicates the inherent reactivity of Na_2_DMGO under electrochemical charging conditions. Conversely, the appearance of a distinct N=O peak at 1426 cm^−1^ and N=O peak at 1590 cm^−1^ in the course of charging unequivocally confirms the formation of new chemical species, offering compelling evidence for the Na^+^ release mechanism of Na_2_DMGO.^27^ A characteristic peak corresponding to DMODO was detected at *m*/*z* 115 ([M + H]^+^) (Fig. 2g). The XRD patterns and Raman spectra of the Na_2_DMGO electrode, collected at various stages of Na^+^ extraction, provide comprehensive insights into the material's structural evolution upon desodiation (Fig. S19 and S20). The analysis revealed the complete disappearance of the Raman and XRD peaks associated with Na_2_DMGO, thereby unequivocally validating its full decomposition. In the FT-IR spectra of the electrolyte collected during the charging process (Fig. S21), the pristine electrolyte shows no signal of the additive. However, upon charging to 3.5 V and further to 4.2 V, a distinct N–O vibration peak emerges in the electrolyte. This provides direct compositional evidence that the solid-phase Na_2_DMGO additive successfully decomposes to release Na^+^ and transforms into DMODO dissolved in the electrolyte. The structural identity of the DMODO byproduct was further confirmed by ^1^H NMR spectroscopy (Fig. S22) and ^13^C NMR spectroscopy (Fig. S23). The spectrum, collected in CDCl_3_ characterized by the solvent peak at 7.26 ppm, clearly exhibits two distinct, sharp singlets in the high-field region at approximately 2.15 ppm and 2.30 ppm. These two peaks are unequivocally assigned to the protons of the two –CH_3_ directly attached to the furoxan heterocycle. In ^13^C NMR spectroscopy, two separate signals emerge at approximately 10 ppm and 15 ppm, corresponding to the two –CH_3_. Furthermore, two well-resolved peaks at roughly 144 ppm and 156 ppm are assigned to the two heterocyclic carbons of the furoxan ring. We performed energy calculations on potential intermediates, including DAST and atomic Na to clarify the decomposition mechanism of Na_2_DMGO. As a result of the comparison of energies (Fig. S24), thermodynamic analysis manifests that Na_2_DMGO has a higher energy than the combination of DAST and Na, suggesting a favourable decomposition pathway.

**Fig. 3:**
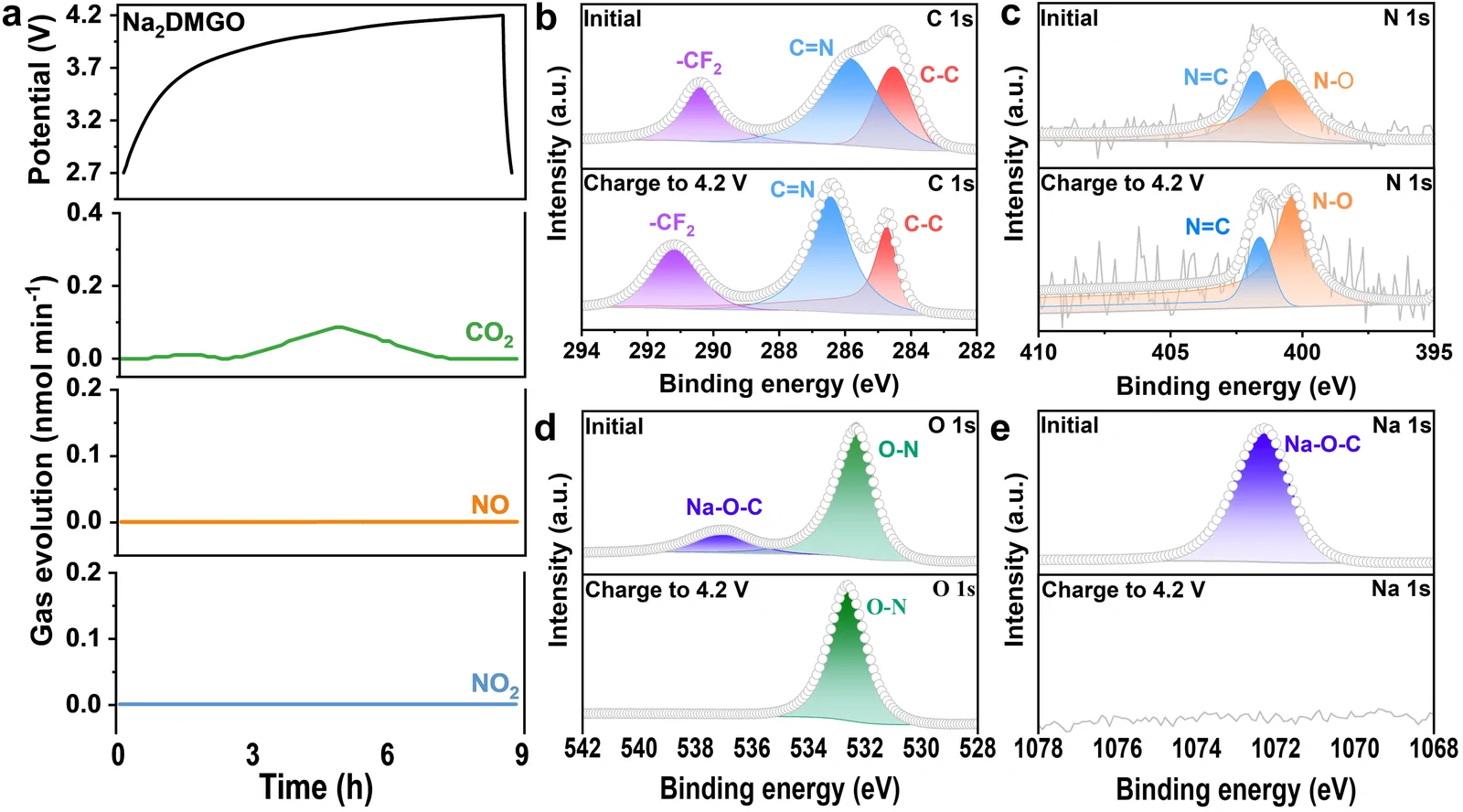
(a) DEMS for Na_2_DMGO. (b) C 1s, (c) N 1s, (d) O 1s, and (e) Na 1s XPS spectra of Na_2_DMGO during the charging process.

DEMS and XPS analyses were conducted on Na_2_DMGO before and after charging to uncover its decomposition mechanism. Accordingly, DEMS (Fig. 3a) showed that no NO_2_ or NO gas was detected. Unlike sodium acetate (AC–Na) for generating C_2_H_6_,^15^ the Na_2_DMGO electrode evolves only CO_2_ derived from battery side reactions. XPS measurements were performed on both pristine and fully charged electrodes to gain detailed insights into the material's electrochemical behavior. The conversion of Na_2_DMGO to DMODO after full charging is dictated by the marked enhancement of the C=N peak at 286.6 eV in the C 1s XPS spectrum (Fig. 3b), in agreement with the comprehensive XPS survey (Fig. S25). Upon full charging, the pronounced reduction of the Na peak at 1072.5 eV unequivocally confirms the efficient extraction of Na^+^. At 4.2 V (Fig. 3c and e), the complete extraction of Na^+^ corresponds to the disappearance of the Na–O peak at 537.1 eV.^28–30^ Additionally, the significant enhancement of the N–O characteristic peak at 400.4 eV in the DMODO product, relative to its initial state in Na_2_DMGO, illustrates the decomposition of Na_2_DMGO.^31–33^ SEM and EDS were employed to examine the electrode morphology and elemental distribution (Fig. S26). The SEM images highlight that the electrode retains structural integrity, while the EDS spectra validate no detectable sodium element residues on the surface after Na_2_DMGO decomposition.

**Fig. 4:**
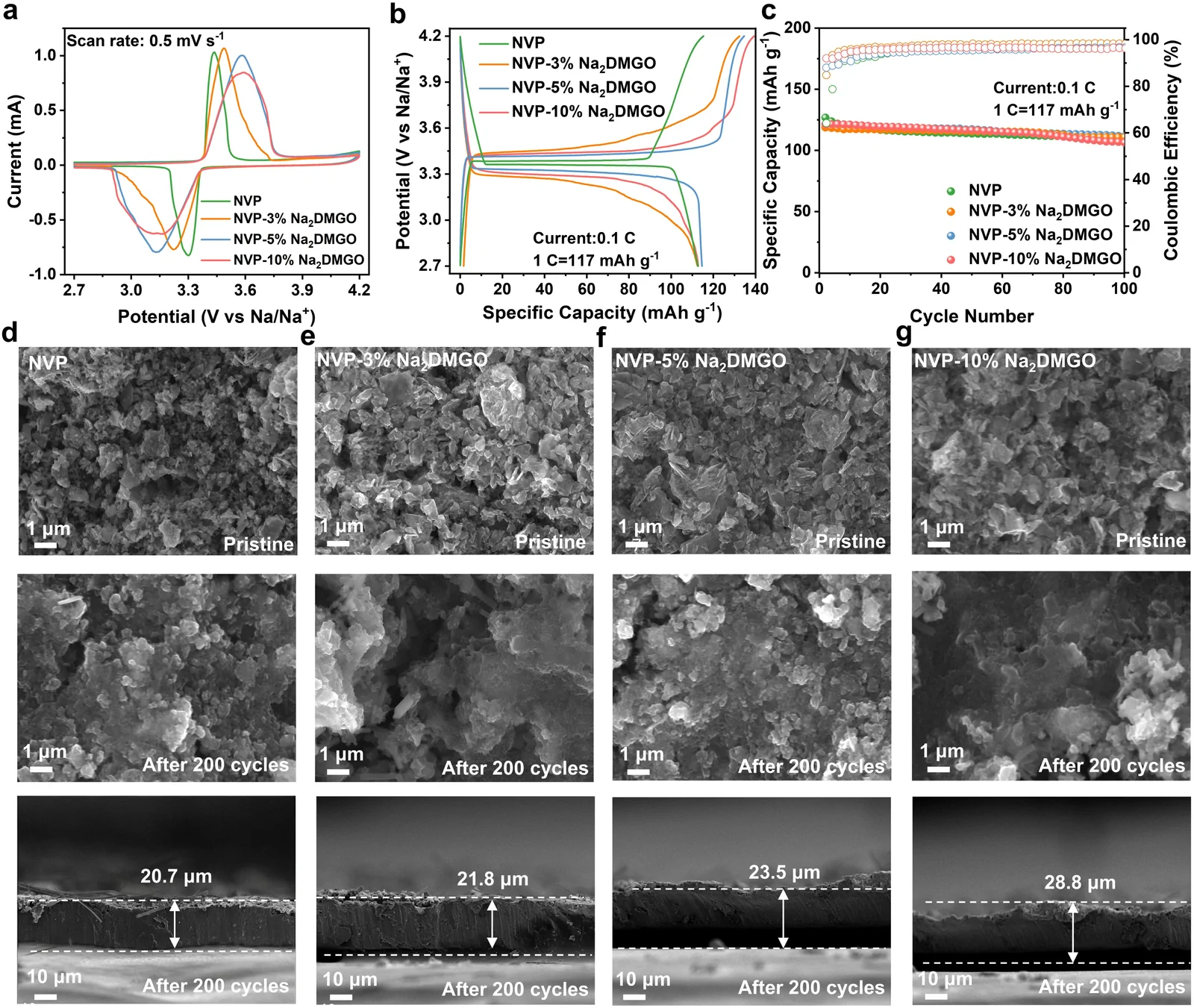
Electrochemical characteristics of the NVP, NVP-3% Na_2_DMGO, NVP-5% Na_2_DMGO, and NVP-10% Na_2_DMGO electrodes in a half-cell: (a) CV curves, (b) GCD curves, and (c) cycling performance. SEM of the electrodes. Top: pristine surface morphology. Middle: surface morphology after 200 cycles. Bottom: cross-sectional SEM images showing cycled thickness for (d) NVP, (e) NVP-3% Na_2_DMGO, (f) NVP-5% Na_2_DMGO, and (g) NVP-10% Na_2_DMGO electrodes.

Half-cells employing a metallic sodium counter electrode were fabricated to elucidate Na^+^ compensation of Na_2_DMGO and assess its impact on the overall electrochemical performance of the NVP cathode. In CV curves, a slight shift in redox potentials is observed with increasing Na_2_DMGO additive content (3–10 wt%), with the oxidation potential shifting positively and the reduction potential shifting negatively (Fig. 4a). This correlation between increased CV peak areas and enhanced initial charging capacity confirms the direct contribution of the Na_2_DMGO additive. Optimal battery performance was achieved at an additive content of 5 wt%. The galvanostatic charge–discharge profiles of NVP electrodes containing varying amounts of Na_2_DMGO (3–10 wt%) were evaluated (Fig. 4b). At 0.1C (1C = 117 mAh g^−1^), the pristine NVP electrode delivers initial charge and discharge capacities of 115 and 113 mAh g^−1^. The initial charge/discharge capacities of the NVP electrode incorporating 3–10 wt% Na_2_DMGO additives are measured to be 132/112, 134/114, and 139/108 mAh g^−1^, respectively. The additional charging capacity of 17–24 mAh g^−1^ can be attributed to the inclusion of 3–10 wt% Na_2_DMGO additives. In terms of rate performance, the pristine NVP, NVP-3% Na_2_DMGO, and NVP-5% Na_2_DMGO electrode delivered similar capacities (∼107 mAh g^−1^) at 4C (Fig. S27). By comparison, the NVP-10% Na_2_DMGO electrode delivered a marginally lower specific capacity of approximately 100 mAh g^−1^ at 0.1C. The electrochemical stability of NVP in half-cells was demonstrated by the stable capacities of 106–111 mAh g^−1^ maintained by all electrodes after 100 cycles (Fig. 4c), underscoring the positive role of moderate active Na^+^ supplementation. To unequivocally establish that the capacity gain originates from Na^+^ release in Na_2_DMGO and to assess the influence of the decomposition products, we performed control experiments using the precursor H_2_DMGO, as an additive. The comparative CV curves and GCD curves show that adding 5 wt% H_2_DMGO exerts an adverse effect on the NVP cathode, manifested as shifted CV peaks, unstable capacity, and increased impedance (Fig. S28). The post-cycling characterization (Fig. S29) indicates that incorporating H_2_DMGO leads to significant morphological changes in the NVP cathode, which consequently impairs its capacity utilization. We performed *in situ* EIS testing on the NVP electrode with varying additions of Na_2_DMGO (Fig. S30). It is evident that, with the exception of the 10% Na_2_DMGO composite, the other three electrodes exhibit similar impedance characteristics, showing a gradual decrease in impedance. This observation reveals that introducing an appropriate amount of Na_2_DMGO is unlikely to impede charge transfer kinetics at the electrode interface.

Furthermore, we analyzed the composition of the electrolyte extracted from the cells after long-term cycling. In the Raman spectra (Fig. S31), the characteristic peaks of the electrolyte with the Na_2_DMGO additive (from 3% to 10%) manifest no significant shifts or emergence of unexpected parasitic peaks compared to the pristine electrolyte. Throughout prolonged cycling, DMODO does not trigger continuous parasitic reactions or accelerate the degradation of the bulk electrolyte solvents and salts. As the expected outcome of Na_2_DMGO oxidative decomposition, the presence of DMODO in the cycled electrolyte is confirmed by the N–O bond in the FT-IR spectra (Fig. S32). The results of the spectroscopic and morphological analyses verify that at the optimal dosage (5%), the presence of DMODO in the electrolyte is chemically benign and works synergistically to maintain a highly stable electrode/electrolyte interface without continuous detrimental accumulation. Transmission electron microscopy (TEM) characterization reveals a dense morphology in the pristine material (Fig. S33). Upon charging to 4.2 V, the structure of NVP remains intact. Similarly, when discharged to 2.7 V, the material maintains its structural integrity without observable damage. These findings suggest that the structural evolution of NVP remains largely unaffected by the incorporation of Na_2_DMGO. The morphological features and elemental mappings of NVP-based electrodes, containing different dosages of Na_2_DMGO, were characterized *via* SEM and EDS mapping (Fig. S34) to evaluate the structural integrity before and after electrochemical cycling. Initially the pristine NVP electrode (Fig. 4d) illustrates a relatively compact but aggregated surface, manifesting limited surface uniformity. Upon incorporation of Na_2_DMGO, especially at 5 wt% (Fig. 4f), the electrode surface becomes more uniformly distributed with finer and more interconnected particles, suggesting improved initial morphology conducive to stable cycling. After 100 cycles, the NVP electrode without an additive presents severe surface deterioration and large cracks, indicating unstable SEI formation. The morphology of the NVP-3% Na_2_DMGO electrode (Fig. 4e) reveals an incomplete surface coating and a concomitant increase in porosity across the electrode architecture. NVP-5% Na_2_DMGO maintains an intact and dense structure, with minimal degradation, implying enhanced interfacial stability. In contrast, NVP-10% Na_2_DMGO (Fig. 4g) depicts overgrowth of surface crystals and morphological inhomogeneity, likely caused by excessive additive decomposition. These observations confirm that NVP-5% Na_2_DMGO offers the optimal balance between Na^+^ compensation and structural stability, effectively preserving electrode integrity throughout long-term cycling.

Cross-sectional SEM and EDS mapping were conducted on NVP cathodes with varying Na_2_DMGO (Fig. 4d, f and S35), aimed at directly verifying its distribution uniformity throughout the electrode thickness and the resulting influence on the electron/ion conduction network. As the Na_2_DMGO content increased from 0% to 10%, the electrode thickness increased progressively from 20.7 µm (NVP), 21.8 µm (NVP-3 Na_2_DMGO), and 23.5 µm (pristine NVP-5 Na_2_DMGO) to 28.8 µm (NVP-10% Na_2_DMGO). Crucially, the cross-sectional EDS mapping unequivocally confirms the homogeneous distribution of C, N, O, and Na elements throughout the entire thickness of the electrode. The thicker electrode slightly prolongs Na^+^ diffusion pathways and increases inter-particle resistance. The morphological evolution highly aligns with the kinetic behavior deduced from the EIS results (Fig. S30). Among the investigated electrodes, the one modified with 5 wt% Na_2_DMGO exhibits the most advantageous interfacial characteristics. In comparison, an excessive additive content of 10 wt% causes uneven surface deposits and elevated impedance, while an insufficient 3 wt% loading fails to fully supply enough Na^+^, both resulting in poorer cycling stability. We performed XPS on cathodes with varying additive contents (3%, 5%, and 10% Na_2_DMGO) after 200 cycles (Fig. S36), to conclusively address whether DMODO adversely affects electrode/electrolyte interface stability or accumulates harmfully. Crucially, signal intensity remains negligible or completely absent near the characteristic N–O/N–C binding energy regions in the high-resolution N 1s spectra. This near-zero N signal response directly confirms that DMODO effectively dissolves into the electrolyte.

**Fig. 5:**
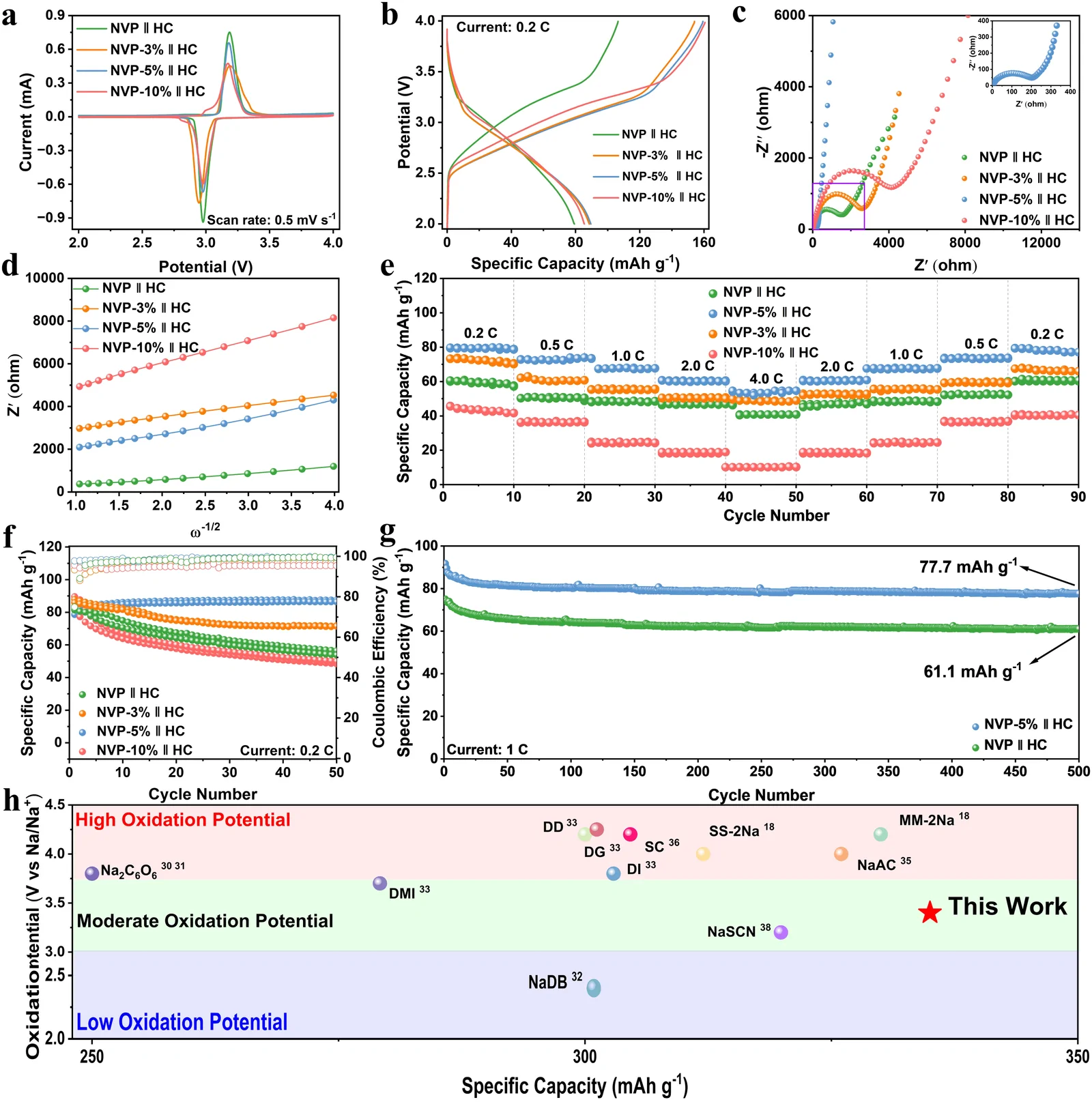
Electrochemical characteristics of the NVP, NVP-3% Na_2_DMGO, NVP-5% Na_2_DMGO, and NVP-10% Na_2_DMGO electrodes in full cells: (a) CV curves, (b) GCD curves, (c) EIS Nyquist plots, (d) *Z*′–*ω*^−1/2^, (e) rate capability, (f) cycling performance, and (g) long-term cycling performance. (h) Comparison of the electrochemical performance of the organic presodiation additives.

Comprehensive structural analyses and electrochemical evaluations of the NVP cathode and hard carbon (HC) anode are provided (Fig. S37–S42). HC delivers an initial charge/discharge capacity of 298/370 mAh g^−1^ at 0.2C under half-cell conditions, with a low ICE of 80.5% (Fig. S39). The low ICE is attributed to the consumption of active Na^+^, resulting from both the formation of the SEI and the irreversible trapping of Na^+^ within the defects and micropores of the HC. In an effort to mitigate the initial Na^+^ loss in SIBs, full cells were fabricated with NVP cathodes, commercial HC anodes, and varying amounts (0–10 wt%) of Na_2_DMGO as a presodiation additive. The 5 wt% full cell exhibits a CV peak area closely mirroring that of pristine NVP (Fig. 5a). This observation suggests that the intrinsic redox signatures of the NVP cathode are largely maintained upon the incorporation of Na_2_DMGO. To assess the efficacy of Na_2_DMGO in enhancing the ICE of the NVP cathode, the GCD profiles of the full cells were evaluated (Fig. 5b). The NVP-HC system indicates an initial discharge capacity of 79 mAh g^−1^ achieving a coulombic efficiency of 74.5% and an energy density of 117 Wh kg^−1^ at a current density of 0.2C (based on the mass of the active material only). The NVP full cells with 3%, 5%, and 10% Na_2_DMGO deliver charge/discharge capacities of 154/88, 159/89, and 160/85 mAh g^−1^, significantly surpassing those of pristine NVP. With the incorporation of 3, 5, and 10 wt% Na_2_DMGO, the corresponding energy densities increase to 130, 131.5, and 125.6 Wh kg^−1^, respectively. In comparison, the addition of 5 wt% Na_2_DMGO is identified as the optimal dosage.

We disassembled the full cells at the fully charged state (4.0 V), and systematically evaluated the surface chemistry of the cycled HC anodes using XPS (Fig. S43). Crucially, the O 1s spectra of both electrodes exhibit a singular, highly symmetric peak at ∼533.3 eV, which is exclusively assigned to the Na–O/Na–O–C species within the SEI components. Furthermore, the anode demonstrates significantly enhanced intensities for Na–F (F 1s), Na–O (O 1s), and CO_3_^2−^ (C 1s and O 1s) peaks. This directly proves that the extra sodium flux was successfully utilized to construct a robust, inorganic-rich SEI layer. We have incorporated this comprehensive discussion, along with XPS, into the revised manuscript. In addition, we characterized the cycled HC anodes using high-resolution transmission electron microscopy (HRTEM), scanning transmission electron microscopy (STEM), and the corresponding EDS elemental mapping. The HRTEM image of the HC anode cycled in the pristine full cell (Fig. S44) reveals a highly non-uniform and thin SEI layer, with its thickness fluctuating widely between 4.8 and 15.7 nm. In contrast, the SEI formed on the HC anode cycled in the Na_2_DMGO full cell displays markedly improved thickness, uniformity, and structural integrity, maintaining a highly consistent thickness of approximately 22.9–23.8 nm (Fig. S44b). In the magnified HRTEM view presented in Fig. S44c, obvious lattice fringes are observed, which are locally discernible and slightly curved. As shown in Fig. S45, STEM images and the corresponding EDS elemental mappings of the HC anode cycled in the Na_2_DMGO full cell exhibit strong, homogeneous Na and O signals localized along the perimeter of the carbon backbone, confirming the uniform distribution and enrichment of sodium- and oxygen-bearing inorganic/organic components throughout the derived SEI layer.

EIS elucidates that after the initial charge–discharge cycle (Fig. 5c), the impedance of the cell with 5 wt% Na_2_DMGO is lower than that of cells with 0, 3, or 10 wt% Na_2_DMGO.^34–37^ This behavior is likely on account of the optimal decomposition of 5 wt% Na_2_DMGO into DMODO, which enhances charge transfer kinetics. Although decomposition occurs at other concentrations, 5 wt% ensures more complete utilization under the given conditions. Consequently, the incorporation of the 5 wt% Na_2_DMGO additive significantly enhances the electrochemical performance of the battery system. The steepest slope is observed for the system (Fig. 5d), reflecting its superior electron transfer efficiency and enhanced redox kinetics. The rate capability tests are conducted at rates ranging from 0.2 to 4C. NVP-5% Na_2_DMGO‖HC shows superior rate performance with a specific capacity of 75 mAh g^−1^ restored at 0.2C (Fig. 5e). Therefore, the 5 wt% ratio is the optimal choice, enabling the full cell to deliver maximum performance. Cycling stability assessments were performed for the four full-cell configurations at 0.2C (Fig. 5f). As anticipated, the NVP-5% Na_2_DMGO‖HC full cell achieved a discharge specific capacity of 85 mAh g^−1^ after 50 cycles at 0.2C, markedly surpassing other proportions. The capacity of NVP-10% Na_2_DMGO‖HC with a capacity retention rate of only 60% after 10 cycles, is attributed to the excessive amount of Na_2_DMGO in the cathode. The integration of 5 wt% Na_2_DMGO into the NVP cathode delivers optimal Na^+^ supplementation, simultaneously maximizing the battery's capacity and ensuring superior cycling stability. Comparative long-term cycling tests were conducted for full cells with and without the additive (Fig. 5g). Remarkably, the optimized full cell retains a high capacity of 77.7 mAh g^−1^ at 1C after 500 cycles.

In order to explain the Na^+^ transport properties influenced by the incorporation of the Na_2_DMGO additive, the galvanostatic intermittent titration technique (GITT)^38–41^ was employed to quantify the Na^+^ diffusion coefficient (*D*^GITT^) in the NVP electrode with 0–10 wt% Na_2_DMGO‖HC. The sodium diffusion kinetics were evaluated using GITT profiles for the NVP electrodes with varying Na_2_DMGO contents (0, 3, 5, and 10 wt%) (Fig. S46 and S47). The *D*^GITT^ of the pristine NVP electrode ranges from 10^−8^ to 10^−12^ cm^2^ s^−1^, which marginally exceed the values of 10^−9^ to 10^−15^ cm^2^ s^−1^ observed for the electrodes containing 3–10 wt% Na_2_DMGO.^42–45^ The impact on Na^+^ migration within the NVP electrode remains negligible for Na_2_DMGO contents between 3 and 10 wt%. Compared to the existing organic additives outlined (Fig. 5h), Na_2_DMGO exhibits comprehensive superiority. It delivers an optimal combination of high decomposition kinetics and high irreversible capacity, while simultaneously ensuring remarkable structural stability and the absence of detrimental side reactions. These attributes collectively underscore the promising potential of Na_2_DMGO as an efficient cathode sodium compensation additive. Consequently, Na_2_DMGO emerges as an efficient sodium-donating component capable of markedly enhancing the capacity of next-generation SIBs, offering a promising avenue toward their large-scale advancement.

## Conclusions

In summary, we present Na_2_DMGO as an air-stable and cost-effective presodiation additive for SIBs. The additive is prepared through a one-step substitution process and delivers a near-theoretical capacity of 330 mAh g^−1^. Na_2_DMGO decomposes at 3.4 V, efficiently compensating for the Na^+^ loss caused by SEI formation and side reactions. *In situ* FT-IR and DEMS confirm, for the first time, the complete decomposition of Na_2_DMGO into DMODO, a non-toxic and electrochemically inert product. The decomposition of Na_2_DMGO proceeds without gas evolution and the resulting DMODO byproduct ensures no interference with full cell operation. When 5 wt% Na_2_DMGO was incorporated into the Na_3_V_2_(PO_4_)_3_(NVP) cathode and paired with hard carbon, it markedly improved the overall battery performance, boosting capacity retention from 62% to 85% over 50 cycles. Furthermore, this addition resulted in a 12.4% enhancement in the energy density (based solely on the mass of the active material) of the full cell compared to the additive-free counterpart. With its straightforward synthesis, stable molecular structure, and remarkable electrochemical advantages, Na_2_DMGO holds great promise for improving the energy density and durability of SIBs, paving the way for their practical deployment in large-scale energy storage systems.

## Author contributions

Zhibin Zhao: writing – original draft, investigation, data curation, formal analysis, validation, methodology. Linxin Lv: data curation. Xinlei Xu: formal analysis. Jincheng Sun: formal analysis. Yifan Tong: visualization. Kai Zhang: writing – review and editing, methodology, validation. Weiwei Huang: conceptualization, funding acquisition, writing – review and editing, resources, supervision, visualization.

## Conflicts of interest

The authors declare no conflicts of interest.

## Supplementary Material

SC-017-D6SC04167A-s001

## Data Availability

The data that support the findings of this study are available from the corresponding author, upon reasonable request. The data supporting this article have been included as part of the supplementary information (SI). Supplementary information: experimental procedures, NMR spectra and computational details. See DOI: https://doi.org/10.1039/d6sc04167a.
