# Supplementary material for: Air-stable sodium dimethylglyoxime as a cathode presodiation additive for high-energy-density sodium-ion batteries
Source: Chem Sci. 2026 Jun 30. Online ahead of print. doi: 10.1039/d6sc04167a (PMC13352661; doi:10.1039/d6sc04167a)
Supplement: SC-OLF-D6SC04167A-s001 [file SC-OLF-D6SC04167A-s001.pdf]

Supplementary Information (SI) for Chemical Science.
This journal is © The Royal Society of Chemistry 2026

# Supplementary Information

## Air-Stable Sodium Dimethylglyoxime as a Cathode Presodiation Additive for High-Energy-Density Sodium-Ion Batteries

Zhibin Zhao, [ab] Linxin Lv, [ab] Xinlei Xu, [a] Jincheng Sun, [ab] Yifan Tong, [b] Kai Zhang *[b] and Weiwei Huang *[abc]

[a] Hebei Key Laboratory of Applied Chemistry, Yanshan University, Qinhuangdao 066000, P. R. China

[b] Frontiers Science Center for New Organic Matter, Key Laboratory of Advanced Energy Materials Chemistry (Ministry of Education), State Key Laboratory of Advanced Chemical Power Sources, College of Chemistry, Nankai University, Tianjin 300071, P. R. China

[c] School of Chemistry, Tianjin Normal University, Tianjin 300071, P. R. China

## 1.Experimental section

### 1.1 Materials

Dimethylglyoxime ($H_2$DMGO, 99%), sodium methoxide ($CH_3ONa$, 99%)anhydrous N-methyl-2-pyrrolidone (NMP, 99.9%), and polyvinylidene fluoride (PVDF) and metal sodium (≥99.7%) were purchased from Shanghai Aladdin Biochemical Technology Co., Ltd. Methanol ($CH_3OH$, 99.5%), and ethyl alcohol (EtOH, 99.7%) were obtained from Tianjin Kemiou Chemical Reagent Co., Ltd. Ketjen Black (ECP-600JD), 1 M $NaPF_6$ in bis(2-methoxyethyl)ether (DIGLYME) electrolytes were purchased from DoDoChem Technology Co., Ltd. Hard carbon (HC) and commercial $Na_3V_2(PO_4)_3$ (NVP, >99.9%) were purchased from Shenzhen Kejing Star Technology Co., Ltd. All chemicals were used without further purification.

### 1.2 Synthesis of Sodium Dimethylglyoxime ($Na_2$DMGO)

Dimethylglyoxime (1.16 g, 10 mmol) was dissolved in anhydrous methanol (20 mL) and Sodium methoxide (1.08 g, 20 mmol) was added to the solution under stirring. The reaction (performed in an Argon filled glove box), was allowed to stir for 24 h at room temperature. The yellow reaction mixture was poured into 100 mL of tetrahydrofuran ether to precipitate the product, followed by filtration, washing with copious amounts of diethyl ether and drying at 60 °C under vacuum. The product Sodium Dimethylglyoxime ($Na_2$DMGO) was obtained (yield=90 %).

## 2. Physicochemical characterizations

The Fourier transformed infrared (FT-IR) spectra were collected through Thermo Scientific Nicolet iS10. The nuclear magnetic resonance ($^1$H NMR) spectrum was obtained by Bruker AscendTM 600 (600 MHz). Raman spectrum was performed with Renishaw InVia. X-ray photoelectron spectroscopy (XPS) was conducted on a Thermo Scientific K-Alpha using a monochromic AlKa source. The scanning electron microscopy (SEM) images and element mapping analysis were conducted using HITACHI SU9600. The powder X-ray diffraction (XRD) was collected on a D-max-2500/PC instrument with Cu Kα X-ray radiation source over the 2θ

range from 5 ° to 60 ° with a speed of 5 ° $min^{-1}$. Differential electrochemical mass spectrometry (DEMS, Linglu QAS 100) was performed to determine the gas components.

### 3. Electrochemical characterization

Electrochemical performances were evaluated with CR2032 coin cells. For electrode preparation, the obtained electrode active material, conductive additive, polyvinylidene fluoride (PVDF) and $Na_2$DMGO according to the specific mass ratio were mixed and ground for 0.5 h in N-methyl-2-pyrrolidone (NMP) as a solvent to prepare a homogeneous slurry. Particularly, for cathode, it is composed of NVP (80 wt.%), SP (10 wt.%) and PVDF (10 wt.%); for anode, it is composed of commercial hard carbon (90 wt.%) and CMC (10 wt.%); for NVP electrode with additive, the mass ratio of $Na_2$DMGO based on all cathode components is 3 wt.%, 5 wt.% and 10 wt.%, respectively, while the ratio of traditional cathode components including $Na_2$DMGO, conductive additive and binder is still 8:1:1. The mass loadings of the cathode was ~4.7 mg $cm^{-2}$, respectively. In the case of the anode, the active mass loading was around 2.2 mg $cm^{-2}$. The cells were assembled in an argon-filled glovebox using 1 M $NaPF_6$ dissolved in diethylene glycol dimethyl ether (Diglyme) as the electrolytes and glass fiber as the separators. For the half cells, the anodes were home-made sodium foils, while those of the full cells were hard carbon electrodes. Galvanostatic charge/discharge tests were conducted with Land BT2000 battery system (Wuhan, China). Cyclic voltammetry (CV) results were collected at 0.5 mV $s^{-1}$ from electrochemical workstation (CHI760E). Before characterizing cycled electrodes, these electrodes would be washed with dimethyl carbonate (DMC) to remove the electrolyte from the electrode surface and dried in vacuum at room temperature. Electrochemical impedance spectroscopy (EIS) was recorded using an AC amplitude of 5 mV within the frequency range of 100 kHz to 0.01 Hz. Gitt results were collected after stabilizing the battery through five cycles, a current pulse of 0.2 C was applied for 10 minutes, followed by a 30 minutes relaxation period to attain equilibrium potential. The diffusion coefficient of $Na^+$ ions ($D^{GITT}$) can be calculated using the following formula:

$$D^{GITT} = \frac{4}{\pi\tau}\left(\frac{m_B V_M}{M_B S}\right)^2\left(\frac{\Delta E_s}{\Delta E_t}\right)^2$$

Where τ is the duration of the current pulse (s), $m_B$ and $M_B$ are the mass (g) and molar mass (g $mol^{-1}$) of the active material, respectively, $V_M$ is the molar volume of the active material ($cm^3$ $mol^{-1}$), S is the geometric surface area of the electrode ($cm^{-2}$), $\Delta E_s$ is the steady-state potential change (V) caused by the current pulse, and $\Delta E_t$ is the total transient potential change (V) during the current pulse (excluding IR drop).

**4. Computational details**

Geometry optimization and frequency analysis were calculated through density functional theory (DFT) method under B3LYP/6-311G (d, p) basic sets by Gaussian 16 program. The calculated results of total density of states (TDOS) were performed by Multiwfn 3.8 programs.

**Table S1.** Details of the $Na_3V_2(PO_4)_3$ || Hard Carbon full-cells.

| Properties | Cathode | Anode | Electrolyte |
|---|---|---|---|
| Proportion | NVP : Super P: PVDF = 80 : 10 : 10 | Hard carbon : CMC=90:10 | 1.0 M $NaPF_6$ in Diglyme |
| Specific capacity | 111 mAh $g^{-1}$ | 300 mAh $g^{-1}$ | / |
| Mass loading | 4.7 mg $cm^{-2}$ | 2.2 mg $cm^{-2}$ | / |

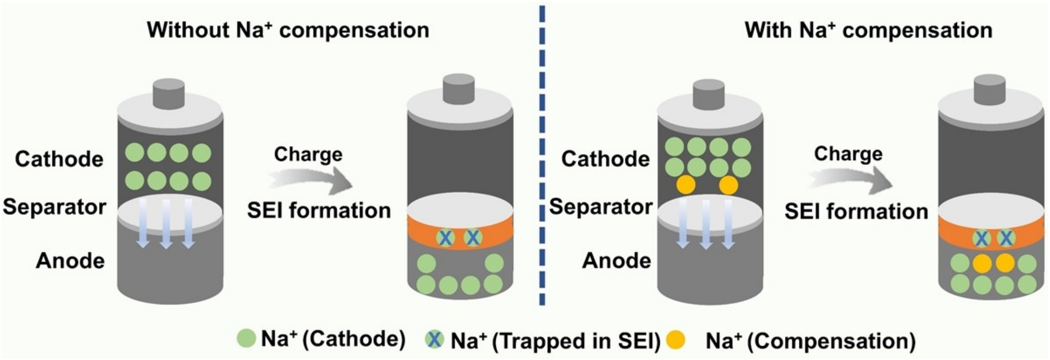


**Figure S1.** Schematic of sodium presodiation additive.

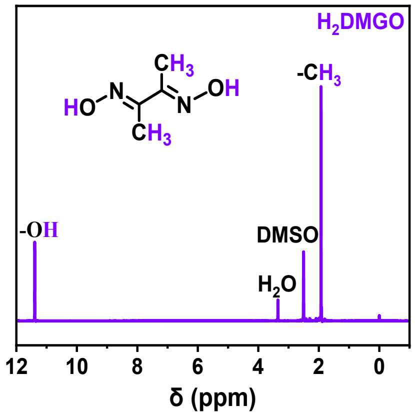


**Figure S2.** $^1$H NMR spectra of $H_2DMGO$.

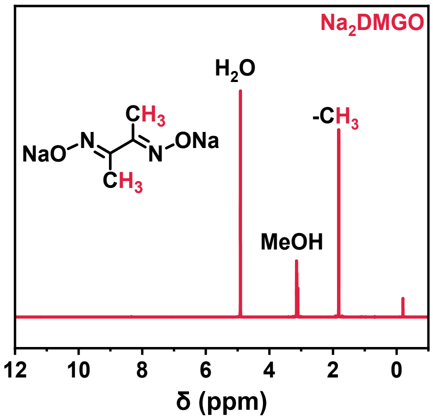


**Figure S3.** $^1$H NMR spectra of synthesized $Na_2DMGO$.

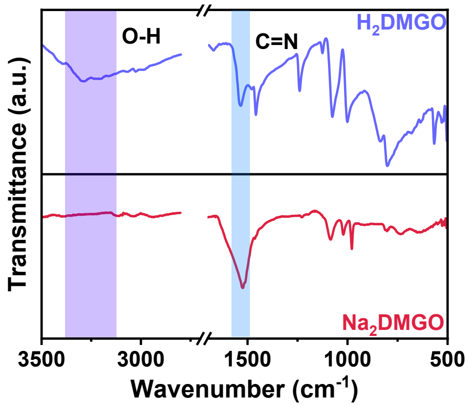


**Figure S4.** FT-IR spectra of $H_2DMGO$ and synthesized $Na_2DMGO$.

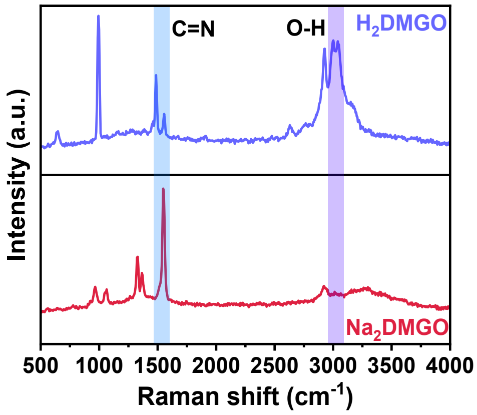


**Figure S5.** Raman spectra of $H_2DMGO$ and synthesized $Na_2DMGO$.

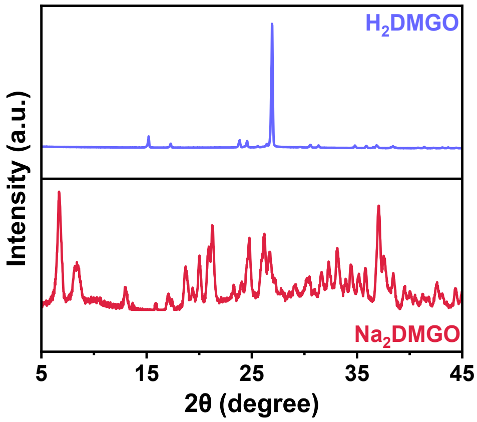


**Figure S6.** PXRD patterns of $H_2DMGO$ and synthesized $Na_2DMGO$.

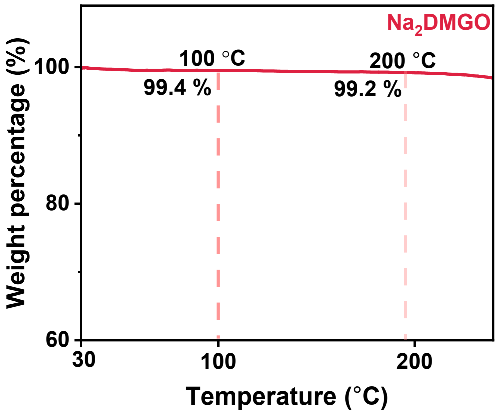


**Figure S7.** TGA of synthesized $Na_2DMGO$.

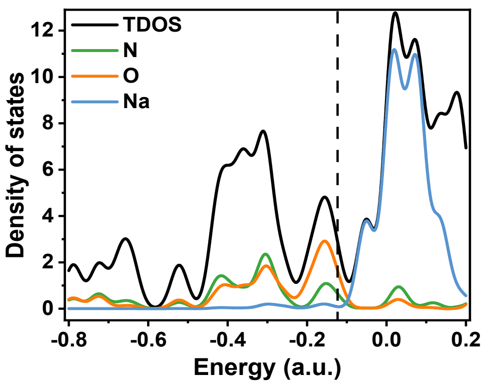


**Figure S8.** The DOS of the $Na_2DMGO$.

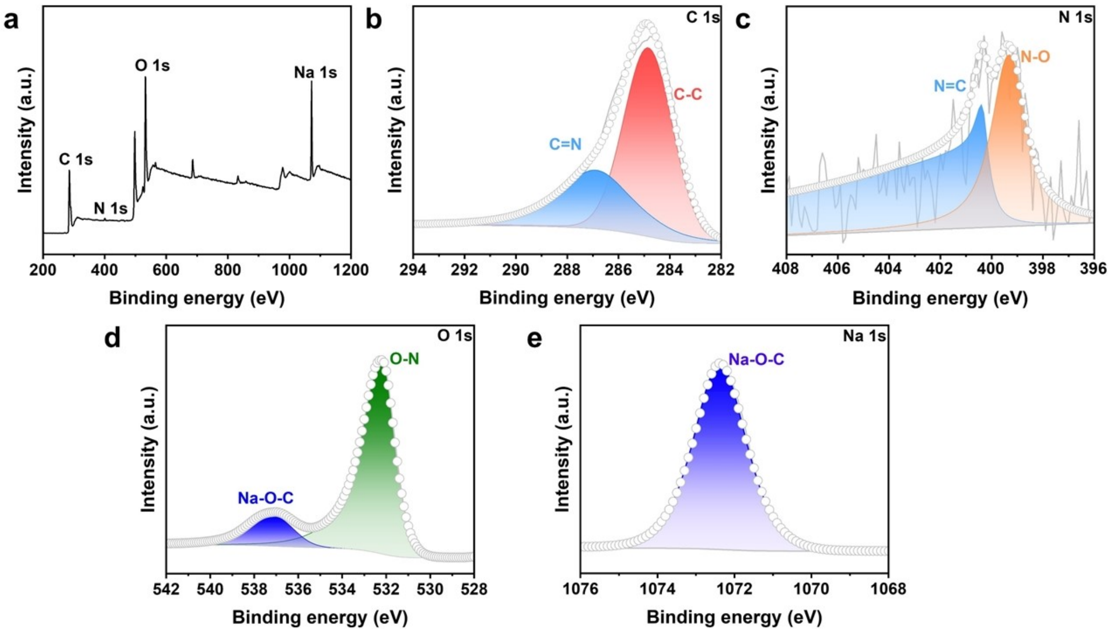


**Figure S9.** (a) XPS survey spectra, (b) C 1s, (c) N 1s, (d) O 1s and (e) Na 1s spectra of $Na_2DMGO$.

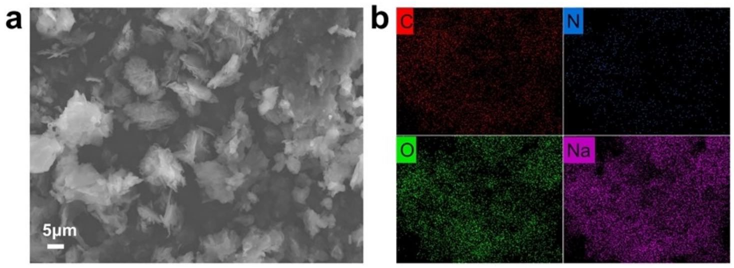


**Figure S10.** (a) SEM of $Na_2DMGO$. (b) EDS mapping of $Na_2DMGO$.

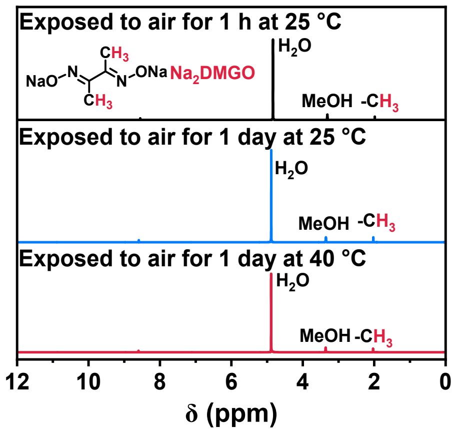


**Figure S11.** $^1H$ NMR spectroscopy of $Na_2DMGO$ powder exposed to air.

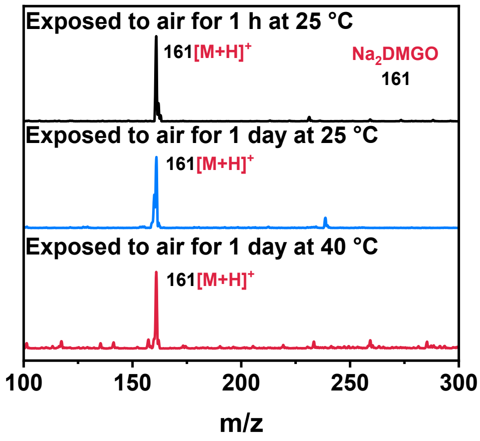


**Figure S12.** MS spectra of $Na_2DMGO$ powder exposed to air.

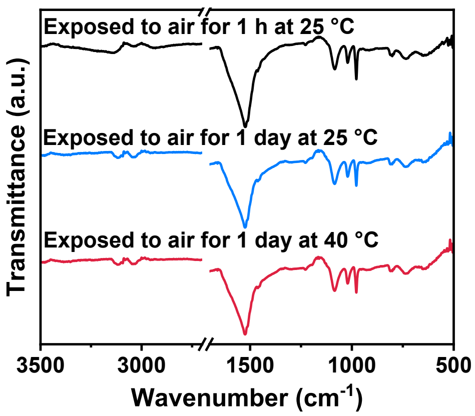


**Figure S13.** FT-IR spectra of $Na_2DMGO$ powder exposed to air.

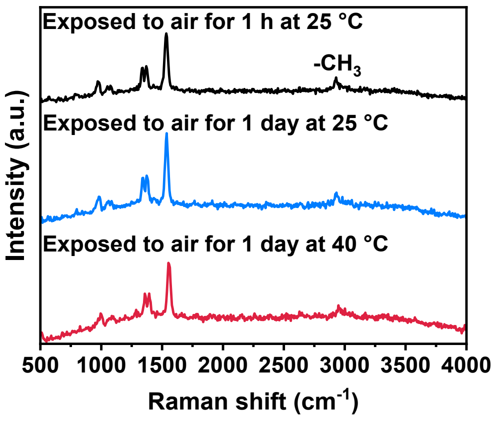


**Figure S14.** Raman spectra of $Na_2$DMGO powder exposed to air.

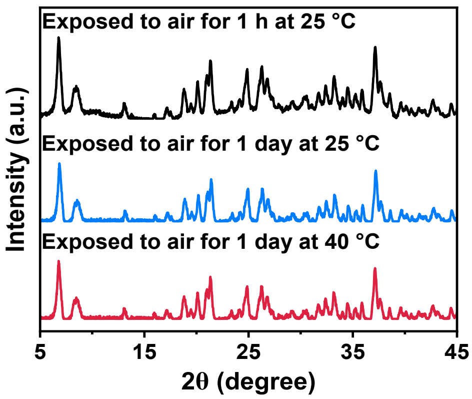


**Figure S15.** XRD patterns of $Na_2$DMGO powder exposed to air.

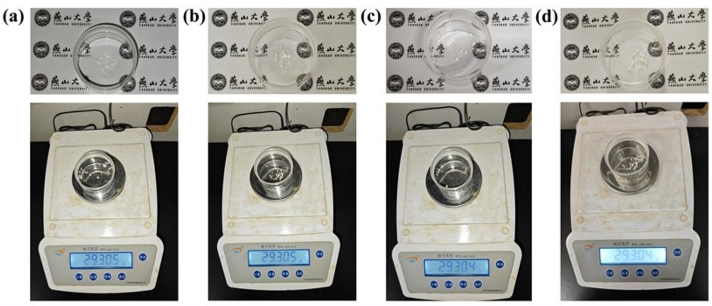


**Figure S16.** Air thermal stability test of $Na_2$DMGO powder at different temperatures.

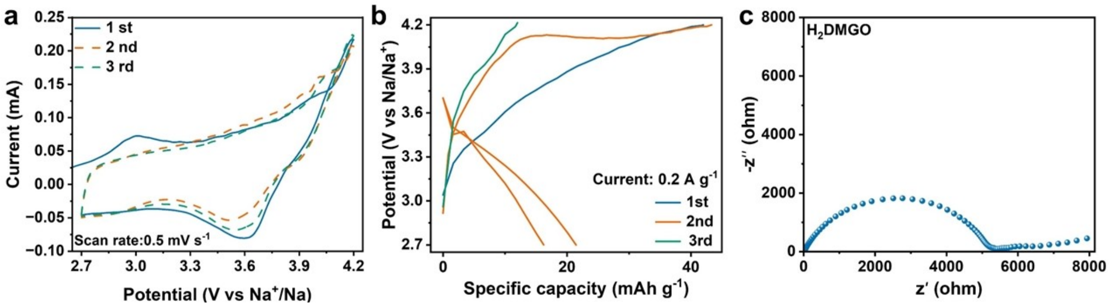


**Figure S17.** (a) CV curves of $H_2DMGO$, (b) GCD curves of $H_2DMGO$, (C) EIS curves of $H_2DMGO$.

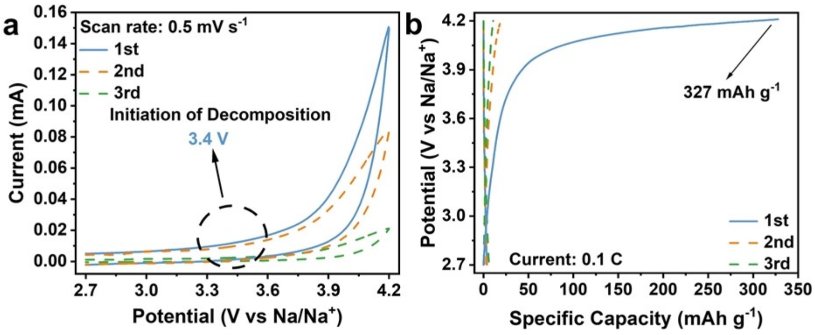


**Figure S18.** (a) CV curves for the $Na_2DMGO$ exposed to air for 1 day at 40 °C. (b) Charge/discharge curves for the $Na_2DMGO$ exposed to air for 1 day at 40 °C.

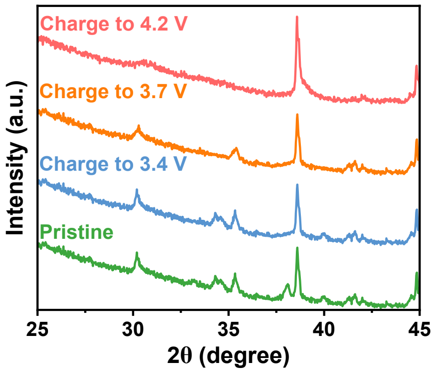


**Figure S19.** XRD patterns of the $Na_2DMGO$ electrode during the charging process.

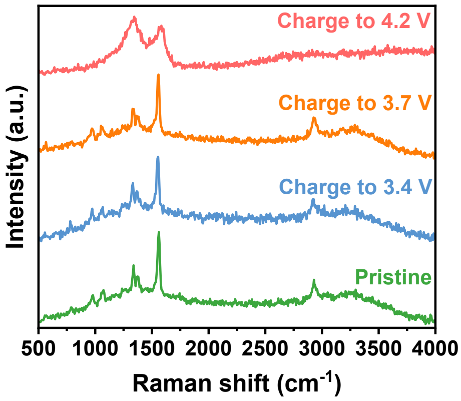


**Figure S20.** Raman spectra of the $Na_2DMGO$ electrode during the charging process.

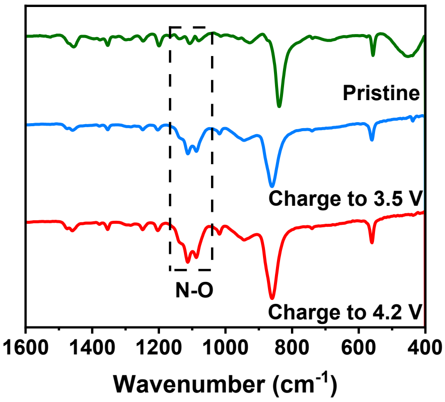


**Figure S21.** FT-IR spectra of the electrolyte during the charging process of $Na_2DMGO$.

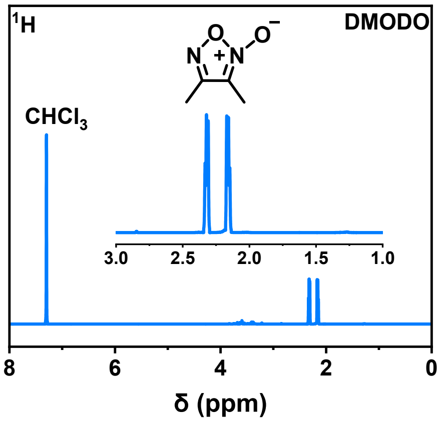


**Figure S22.** $^1H$ NMR of the DMODO in electrolyte during the charging process.

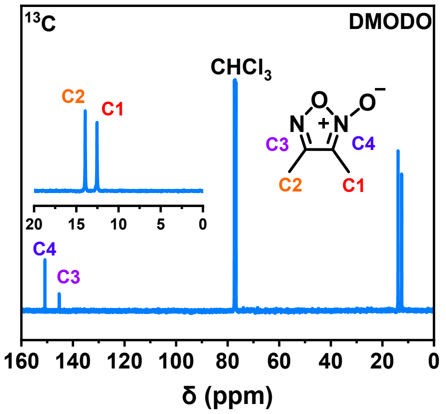


**Figure S23.** $^{13}C$ NMR of the DMODO in electrolyte during the charging process.

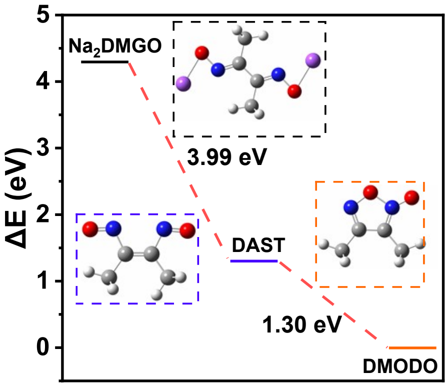


**Figure S24.** The relative energies of the three molecules and the corresponding schematic structural illustrations.

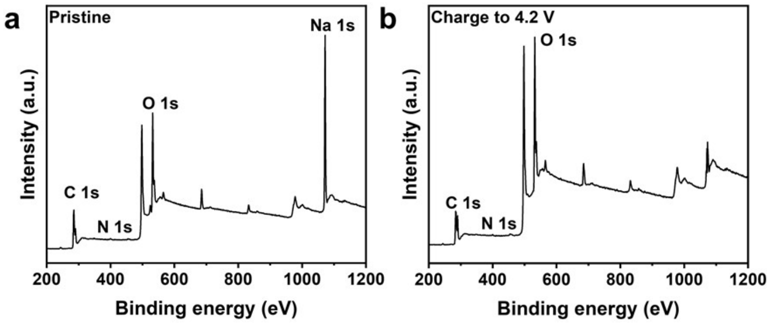


**Figure S25.** XPS spectra of $Na_2DMGO$ electrode during the charging process.

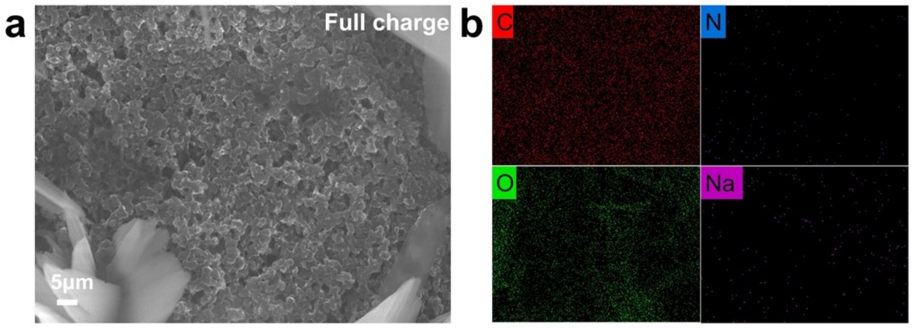


**Figure S26.** SEM images of the desodiated $Na_2DMGO$ electrode and EDS elemental mappings of the desodiated $Na_2DMGO$ electrode.

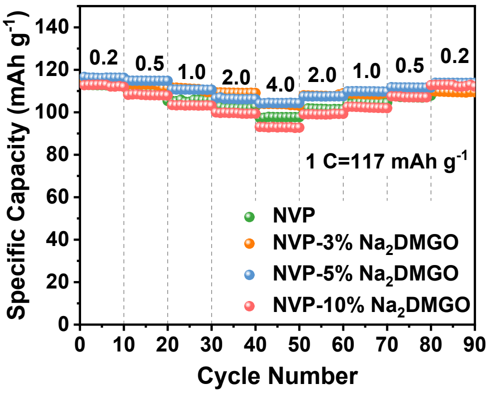


**Figure S27.** Rate performance of the NVP, NVP-3% $Na_2DMGO$, NVP-5% $Na_2DMGO$, and NVP-10% $Na_2DMGO$ electrode in half-cell.

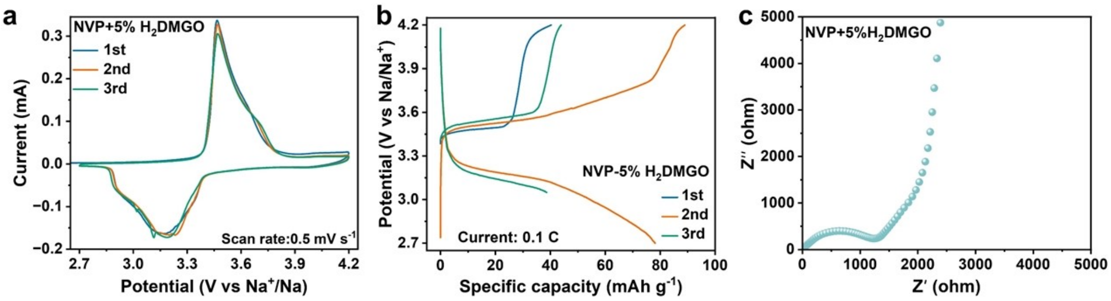


**Figure S28.** (a) CV curves of NVP+5%$H_2DMGO$, (b) GCD curves of NVP+5%$H_2DMGO$, (C) EIS curves of NVP+5%$H_2DMGO$.

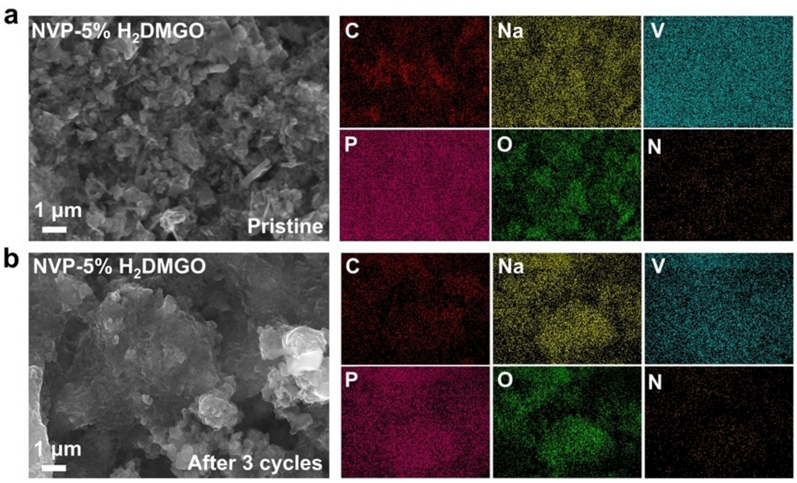


**Figure S29.** (a) SEM images and EDS mapping images of pristine NVP-5%$H_2$DMGO, (b) SEM images and EDS mapping images of NVP-5%$H_2$DMGO after 3 cycles.

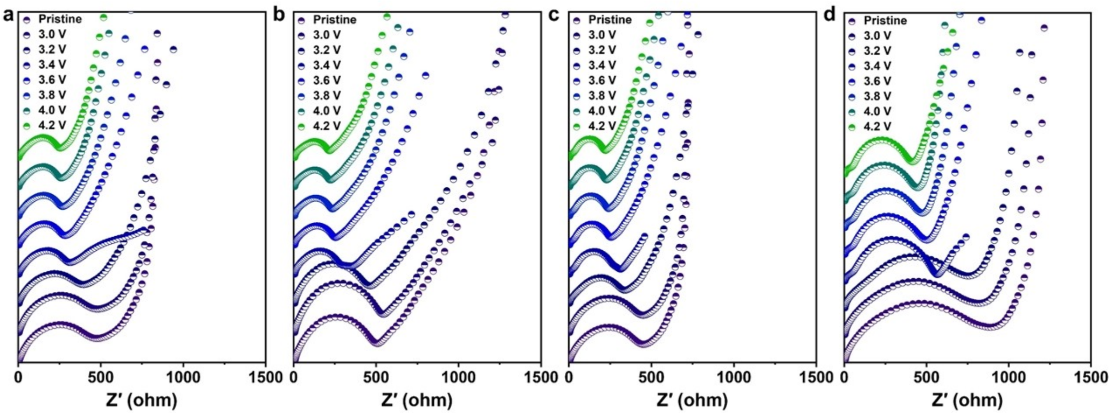


**Figure S30.** In-situ EIS curves of (a) NVP, (b) NVP-3% $Na_2$DMGO, (c) NVP-5% $Na_2$DMGO and (d) NVP-10% $Na_2$DMGO electrodes from pristine charge to 4.2V.

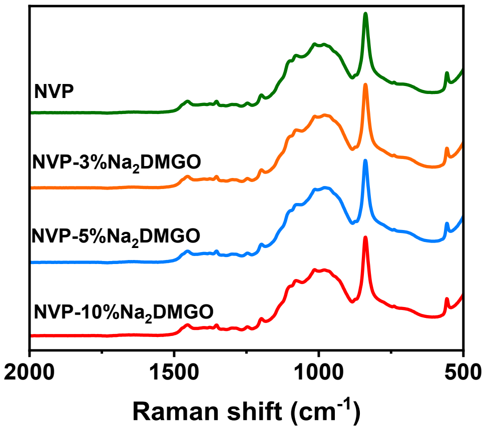


**Figure S31.** Raman spectra of the electrolyte during the charging process.

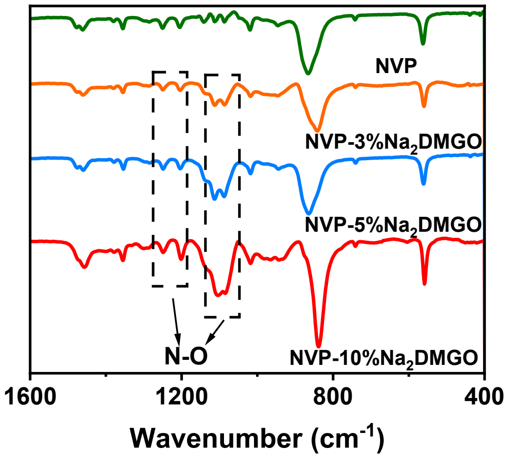


**Figure S32.** FT-IR spectra of the electrolyte during the charging process.

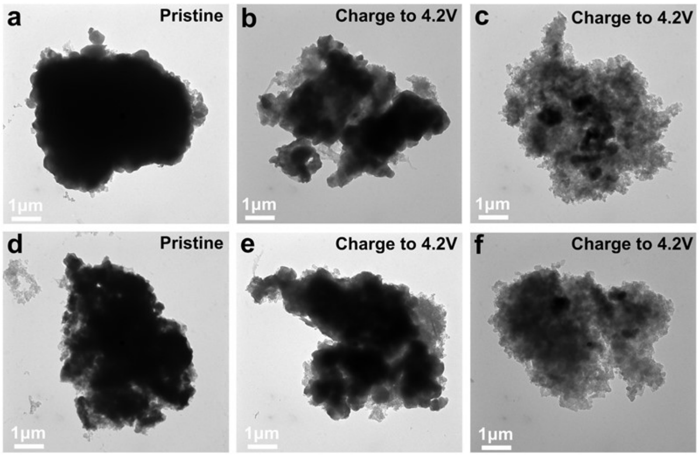


**Figure S33.** TEM images of the NVP-5%$Na_2$DMGO electrode during the charge/discharge process.

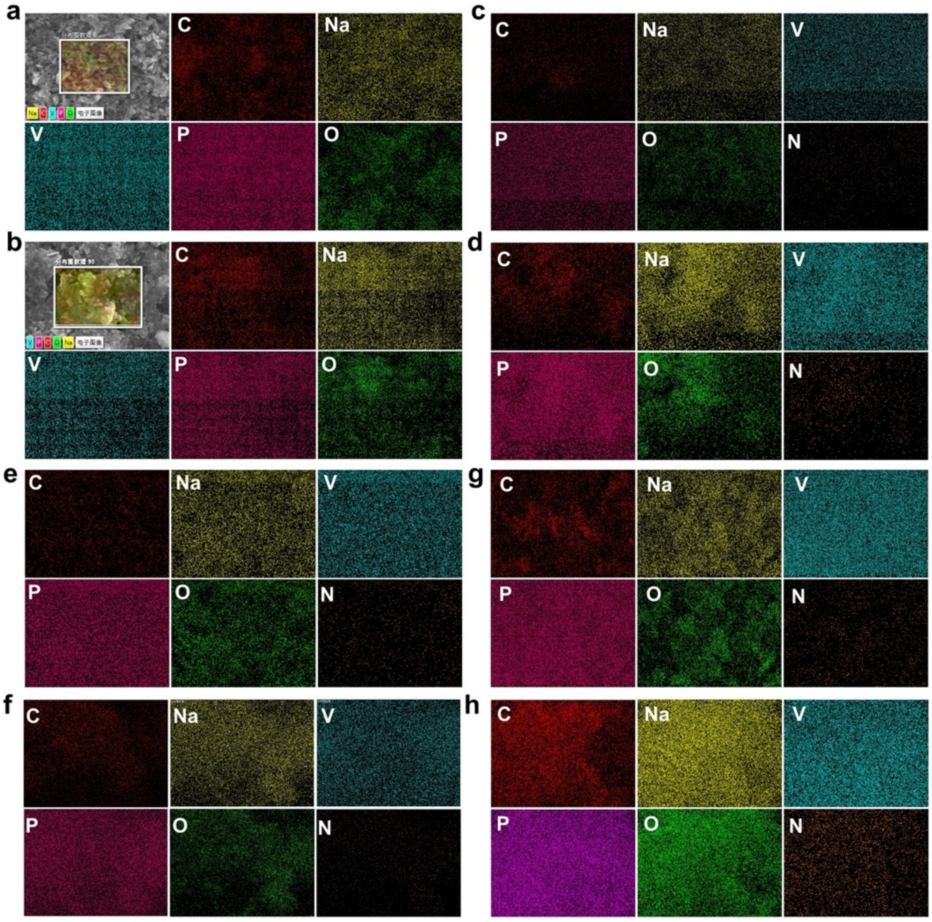


**Figure S34.** Pristine EDS mapping images of (a) NVP, (c) NVP-3% $Na_2$DMGO, (e) NVP-5% and (g) NVP-10% $Na_2$DMGO electrodes. EDS mapping images of (b) NVP, (d) NVP-3% $Na_2$DMGO, (f) NVP-5% $Na_2$DMGO and (h) NVP-10% $Na_2$DMGO electrodes after 200 cycles.

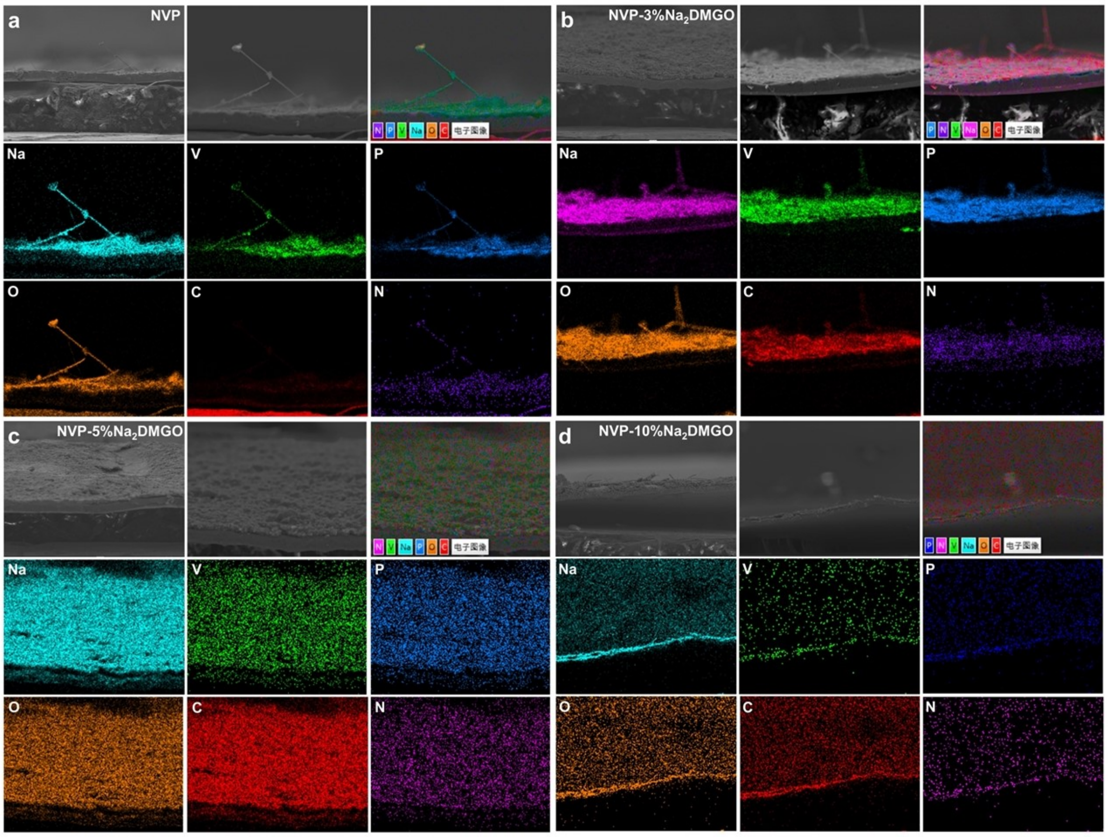


**Figure S35.** Cross-sectional EDS mapping of (a) NVP, (b) NVP-3% $Na_2$DMGO, (c) NVP-5% $Na_2$DMGO and (d) NVP-10% $Na_2$DMGO electrodes.

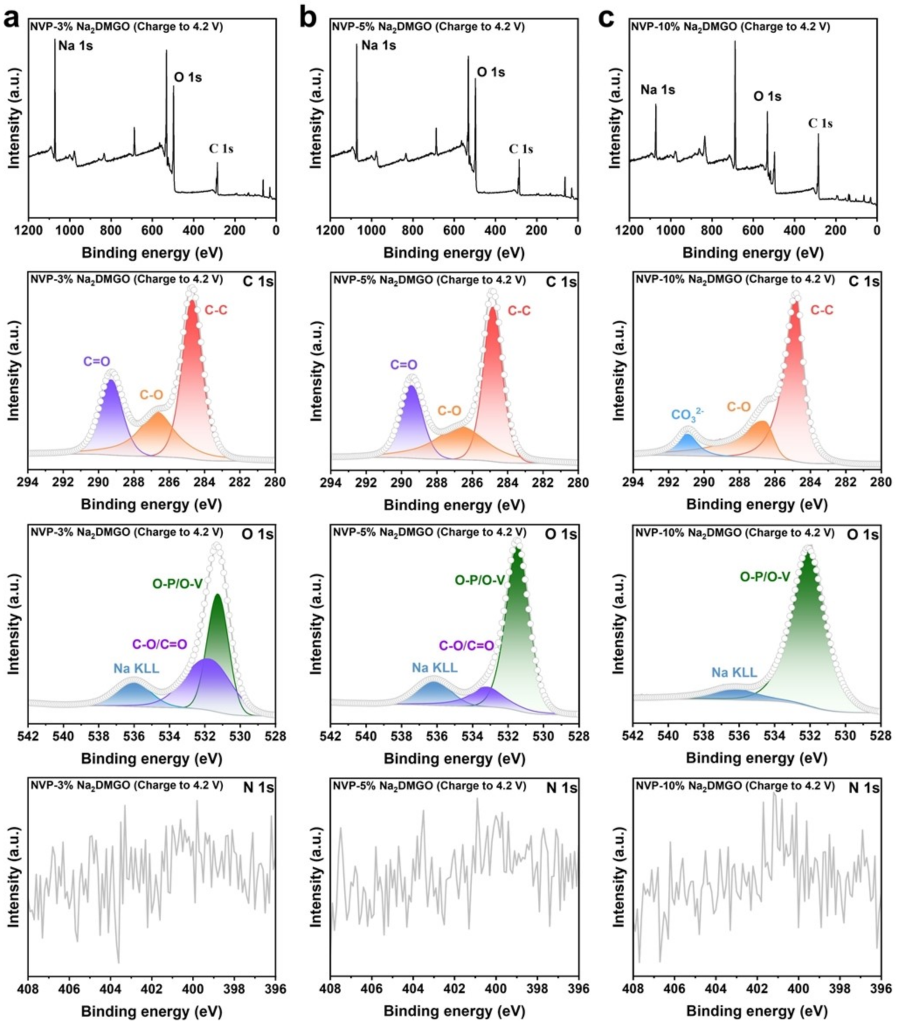


**Figure S36.** XPS spectra of (a) NVP-3% $Na_2$DMGO, (b) NVP-5% $Na_2$DMGO and (c) NVP-10% $Na_2$DMGO electrodes after 200 cycles.

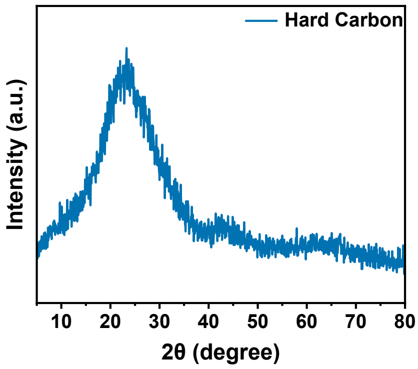


**Figure S37.** XRD patterns of Hard Carbon.

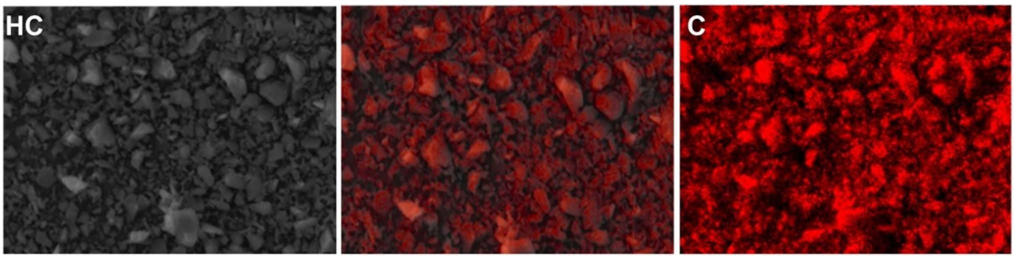


**Figure S38.** SEM and EDS mapping of Hard Carbon.

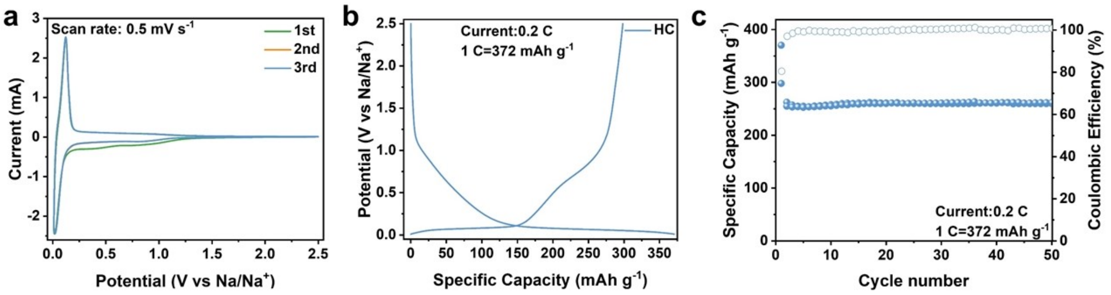


**Figure S39.** (a) CV of Hard Carbon. (b) GCD of Hard Carbon. (c) Cycling performance of Hard Carbon.

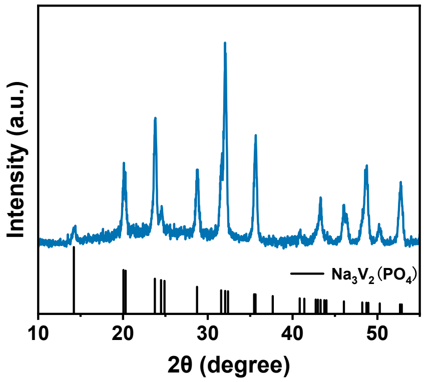


**Figure S40.** XRD patterns of $Na_3V_2(PO_4)_3$.

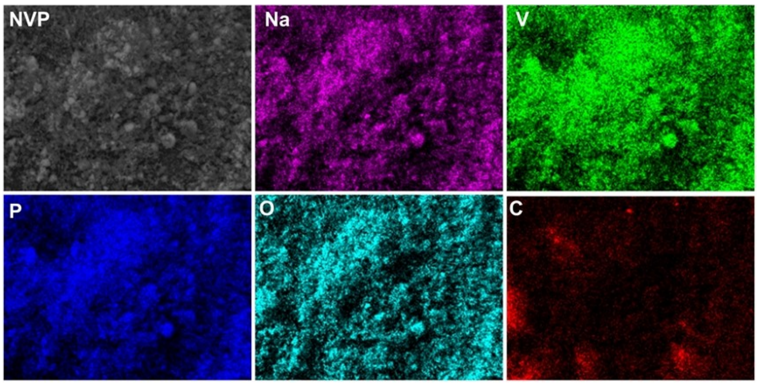


**Figure S41.** SEM images and elemental mapping of $Na_3V_2(PO_4)_3$.

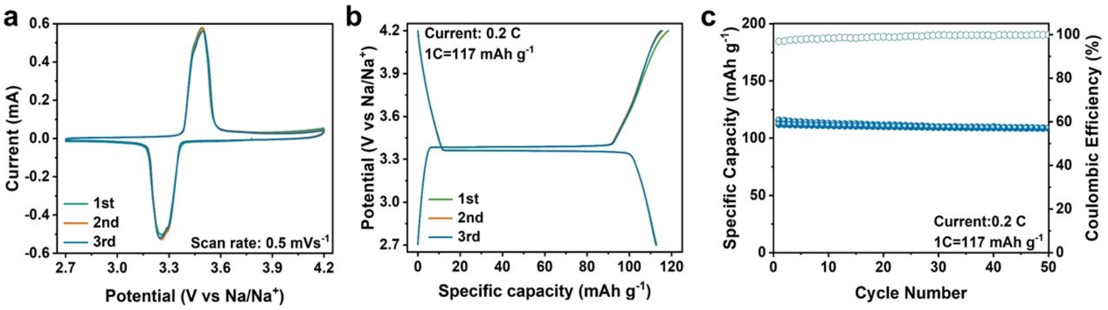


**Figure S42.** (a) CV curve of $Na_3V_2(PO_4)_3$. (b) GCD curve of $Na_3V_2(PO_4)_3$. (c) Cycling performance of $Na_3V_2(PO_4)_3$.

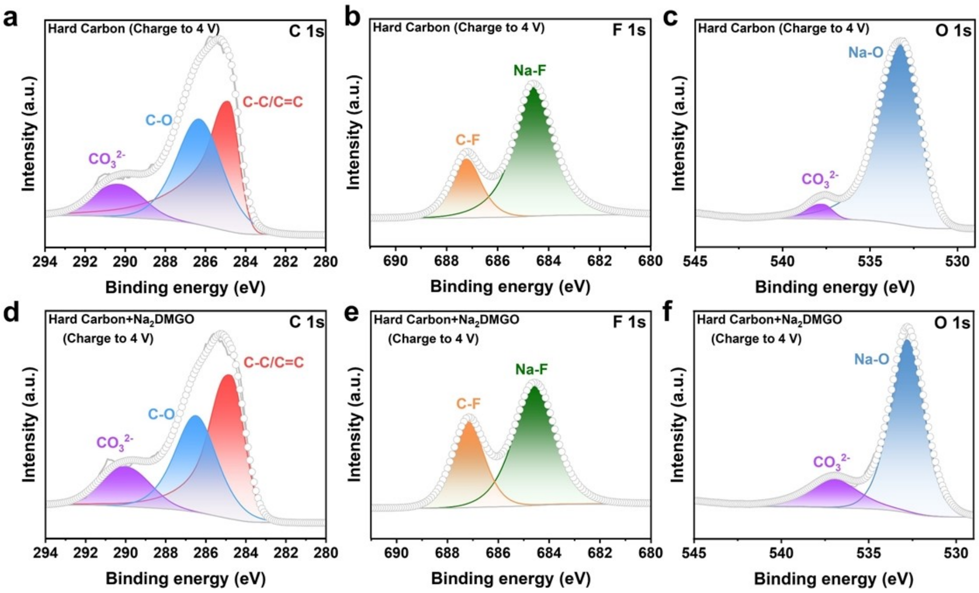


**Figure S43.** (a) C 1s, (b) F 1s, and (c) O 1s spectra of the HC anode cycled in the full cell. (d) C 1s, (e) F 1s, and (f) O 1s spectra of the HC anode cycled in the $Na_2$DMGO full cell.

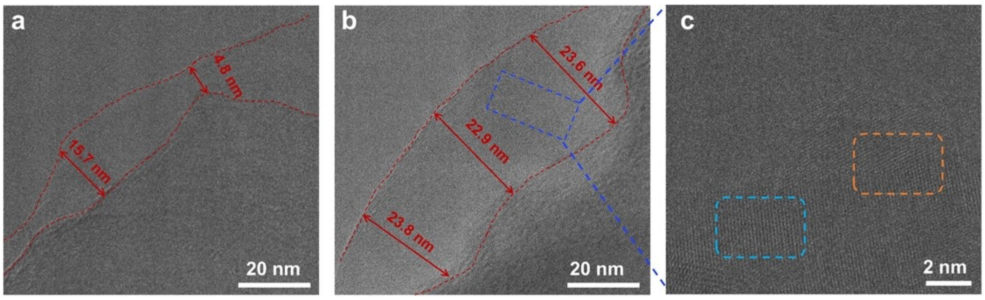


**Figure S44.** (a) HRTEM images of the HC anode cycled in the pristine full cell, (b-c) HRTEM images of the HC anode cycled in the $Na_2$DMGO full cell.

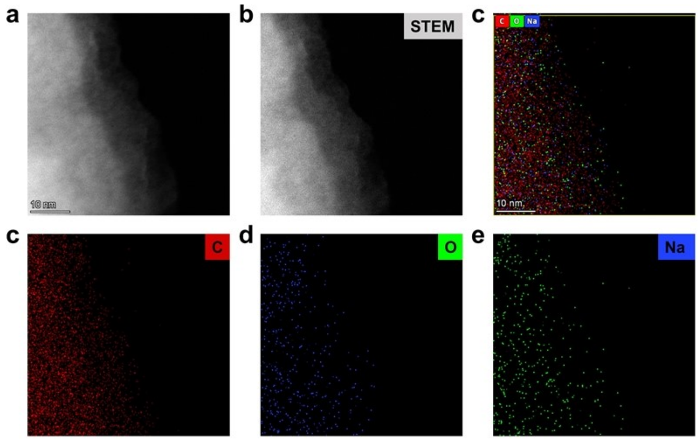


**Figure S45.**EDS mapping of HC anode cycled in the $Na_2$DMGO full cell.

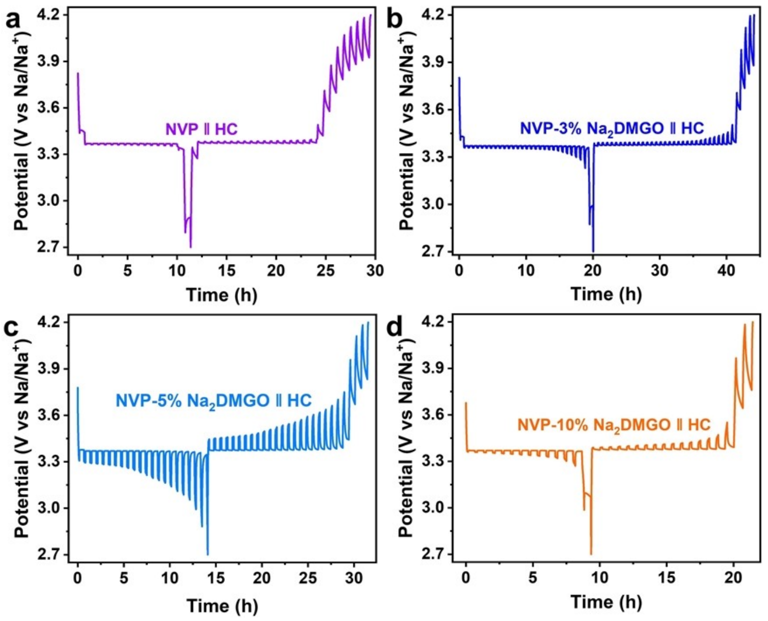


**Figure S46.** (a-d) Charge/discharge profiles via the GITT technique of NVP-$Na_2$DMGO || HC full cell.

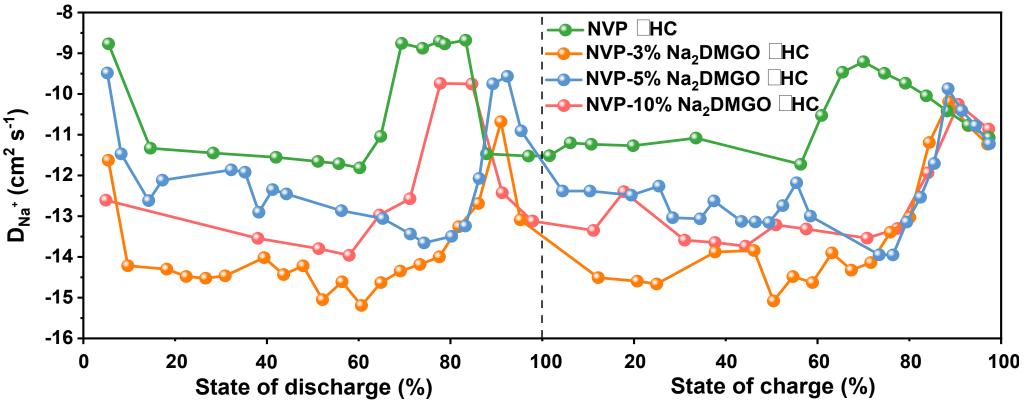


**Figure S47.** The corresponding $Na^+$ diffusion coefficients of NVP-$Na_2$DMGO || HC full cell.

**References**

1. X. Lin, J. Liu, X. Li, Q. Zhao, Y. Zhao, Y. Ni and M. Xu, *Energy Storage Mater.*, 2026, **86**, 104981.

2. Q. Zhang, C. Zhao, X. Zhang, C. He and S. Pang, *New J. Chem.*, 2022, **46**, 1489.

3. L. Hu, P. Hu, Q. Zhang, J. Cong, W. Su, Y. Ren, Y. Kong, J. Zhang, Z. Li and Y. Huang, *Energy Storage Mater.*, 2025, **81**, 100450.
